# Polymicrobial brain abscesses: A complex condition with diagnostic and therapeutic challenges

**DOI:** 10.1093/jnen/nlae058

**Published:** 2024-06-14

**Authors:** Frances-Claire Eichorn, Michelle Kameda-Smith, Crystal Fong, Alice K Graham, Cheryl Main, Jian-Qiang Lu

**Affiliations:** Department of Pathology and Molecular Medicine/Diagnostic and Molecular Pathology, McMaster University, Hamilton, Canada; Department of Pediatrics, Baylor College of Medicine, Houston, TX, United States; Department of Radiology/Neuroradiology, McMaster University, Hamilton, Canada; Department of Pathology and Molecular Medicine/Diagnostic and Molecular Pathology, McMaster University, Hamilton, Canada; Department of Pathology and Molecular Medicine/Microbiology, McMaster University, Hamilton, Canada; Department of Pathology and Molecular Medicine/Diagnostic and Molecular Pathology, McMaster University, Hamilton, Canada

**Keywords:** abscess stage, brain abscess, coinfection, lesion heterogeneity, pathogen, pathogenic complexity, polymicrobial

## Abstract

Brain abscesses (BA) are focal parenchymal infections that remain life-threatening conditions. Polymicrobial BAs (PBAs) are complex coinfections of bacteria or bacterial and nonbacterial pathogens such as fungi or parasites, with diagnostic and therapeutic challenges. In this article, we comprehensively review the prevalence, pathogenesis, clinical manifestations, and microbiological, histopathological, and radiological features of PBAs, as well as treatment and prognosis. While PBAs and monomicrobial BAs have some similarities such as nonspecific clinical presentations, PBAs are more complex in their pathogenesis, pathological, and imaging presentations. The diagnostic challenges of PBAs include nonspecific imaging features at early stages and difficulties in identification of some pathogens by routine techniques without the use of molecular analysis. Imaging of late-stage PBAs demonstrates increased heterogeneity within lesions, which corresponds to variable histopathological features depending on the dominant pathogen-induced changes in different areas. This heterogeneity is particularly marked in cases of coinfections with nonbacterial pathogens such as *Toxoplasma gondii*. Therapeutic challenges in the management of PBAs include initial medical therapy for possibly underrecognized coinfections prior to identification of multiple pathogens and subsequent broad-spectrum antimicrobial therapy to eradicate identified pathogens. PBAs deserve more awareness to facilitate prompt and appropriate treatment.

## Introduction

Brain abscesses (BAs) are focal parenchymal infections that can be caused by various pathogens including bacteria, mycobacteria, fungi, and parasites. Reported incidences of BAs range from 0.4 to 0.9 cases per 100 000 population with increased rates in immunosuppressed individuals.^1–3^ Despite advances in the diagnosis and treatment of BAs during the past 20 years, which have led to continued reductions in mortality, BAs remain life-threatening conditions with an overall mortality rate of approximately 15%; higher rates occur in patients who are elderly, present with multiple abscesses, are immunosuppressed and/or harbor an underlying cardiac anomaly.^2–5^ Polymicrobial BAs (PBAs) involve 2 or more infectious microorganisms within the foci; they are complex coinfections with diagnostic and therapeutic challenges due to difficulties in culturing some microorganisms and requirements for more broad-spectrum empiric antimicrobial therapy to eradicate them.^6–11^ PBAs are not uncommon and are prevalent in otogenic and metastatic abscesses.^6–12^ Moreover, they are likely underreported as some microorganisms such as *Toxoplasma gondii*, and fastidious and anaerobic Gram-negative bacteria are difficult to detect by routine techniques including tissue culture and microscopic examination unless additional techniques such as bacterial RNA or DNA sequencing^6–8^^,^^13–15^ and *Toxoplasma* immunohistochemistry (IHC) or its specific PCR are employed.^16^ Missing detection of one or more pathogens in BAs may lead to an inappropriate spectrum in definitive antibiotic treatment, resulting in disease progression. This article aims to comprehensively review PBAs from their prevalence and pathogenesis to clinicopathological features and treatment.

## Literature searching strategy and selection

For this narrative review, we searched PubMed/MEDLINE for publications before November 2023 using the following keywords: “brain abscess(es),” “cerebral abscess(es),” “cerebellar abscess(es),” or “intracranial abscess(es),” combined with (AND in PubMed) the keywords: “polymicrobial,” “coinfection,” “mixed,” “dual infection,” “polybacterial,” “fungal,” or “parasitic.” While a large number of publications were found from these searches, we excluded publications that did not fit the scope of the topic, namely reported cases of (1) pituitary, epidural, and/or subdural abscesses without central nervous system (CNS) parenchymal involvement,^7^^,^^17–20^ (2) brain monomicrobial infection in patients with systemic infection by other pathogen(s) without evidence of CNS involvement,^21^ and (3) brain viral coinfection without nonviral pathogens causing suppurative cerebritis/encephalitis/BA.^22^ Our review included 89 cases with details reported individually in the past 50 years since computed tomography (CT) was first used (Table S1) and 25 noncase report studies including cases without details published in the past 20 years (summarized in Table 1).

**Table 1.** Polymicrobial brain abscesses reported in noncase report studies published between 2003 and 2023.

Study/Reference	Microbiology testing methods	Number (%) of polymicrobial cases	Detected causative pathogens (dominant)
1. Son et al^13^	16S rRNA versus. culture	10 (40) vs. 2 (8)	*Streptococcus* sp., *Fusobacterium* sp., *Klebsiella* sp., *Porphyromonas* sp., *Campylobacter* sp., *Prevotella*, *Morganella* sp.
2. Kameda-Smith et al^3^	Culture	28 (26)	
3. Andersen et al^6^	16S rRNA	23 (56)	*Streptococcus anginosus*, *Parvimonas* sp., *Prevotella* sp., *Fusobacterium* sp., *Aggregatibacter* sp., *Actinomyces* sp., *Campylobacter* sp., *Porphyromonas* sp., *Aggregatibacter* sp.
4. Stebner et al^7^	16S rRNA	30 (65)	*Fusobacterium nucleatum*, *Streptococcus intermedius*, *Prevotella oris*
5. Hansen et al^29^	16S/18S rRNA	18 (50)	*Actinomyces* sp., *Fusobacterium* sp., *Streptococcus* sp., *Campylobacter* sp., *Escherichia* sp., *Dialister.*, *Filifactor* sp., *Alloprevotella* sp., *Aspergillus* sp.
6. Darlow et al^4^	Culture	9 (19)	*Staphlyococcus* sp., *Streptococcus milleri*,
7. Culbreath et al^24^	16S rRNA	1 (5)	*Fusobacterium nucleatum*, *Streptococcus intermedius*, *Capnocytophaga* spp. *Aggregatibacter aphrophilus*
8. Wu et al^25^	Culture	2 (1.5%)	Bacteria (unspecified)
9. Nkamga et al^23^	16S rRNA	1 (9)	*Methanobrevibacter oralis*, *Aggregatibacter actinomycetemcomitans*
10. Laulajainen Hongisto et al^56^	Culture	5 (28)	Bacteria (unspecified)
11. Kommedal et al^28^	16S rRNA	25 (44)	*Aggregatibacter aphrophilus*, *Fusobacterium nucleatum*, *Streptococcus intermedius*
12. Gadgil et al^57^	Culture	5 (17)	
13. Felsenstein et al^11^	Culture	25 (25.8)	
14. Mathis et al^26^	Culture	9 (45)	
15. Al Masalma et al^58^	16S rDNA	19 (37)	*Bacteroides fragilis*, *Streptococcus intermedius*, *Haemophilus aphrophilus*, *Fusobacterium nucleatum*, *Micromonas micros*, *Streptococcus epidermidis*, *Enterobacter cloacae*, *Enterobacter hormaechei*, *Porphyromonas endodontalis*
16. Sarmast et al^59^	Culture	2 (4)	
17. Nathoo et al^46^	Culture	143 (15)	
18. Al Masalma et al^8^	16S rDNA	8 (40)	*Fusobacterium* sp., *Streptococcus* sp., *Prevotella* sp., *Micromonas micros*, *Eikenella corrodens*
19. Su et al^5^	Culture	17 (14)	
20. Pandey et al^60^	Culture	33 (40)	
21. Dashti et al^41^	Culture	5 (10)	
22. Kocherry et al^61^	Culture	6 (27)	*Bacteroides* sp. *Streptococcus hemolyticus* and nonhemolytic streptococci
23. Cavuşoglu et al^62^	Culture	1 (2)	*Bacteroides fragilis*, *Peptococcus* sp.
24. Carpenter et al^63^	Culture	8 (16)	
25. Jansson et al^64^	Culture	7 (15)	*Streptococcus milleri*, *α Streptococcus* sp.

## Prevalence of polymicrobial brain abscesses

PBAs were traditionally thought to be uncommon, as there were only 89 individually reported cases in the past 50 years (Table S1). However, there have been a considerable number of PBA cases described in noncase report observational studies (Table 1). The number of PBA cases has been increasing, as microbiological techniques have advanced with optimized culture media and molecular diagnostics such as PCR, which is often used in detection of pathogens.^6–8^^,^^13^^,^^14^ Many culture-negative cases have had 2 or more pathogens identified by molecular diagnostics such as 16S rRNA PCR-based detection.^7^^,^^14^^,^^15^^,^^23^^,^^24^ The reported prevalences of PBAs are highly variable depending on the study populations, abscess sources, employed microbiology techniques, and prior antimicrobial treatments.^3–8^ In the studies using observational and cultural microbiology techniques, the prevalence varied from 1.5%^25^ to 45%^26^ (Table 1). The prevalence of PBAs among patients with otogenic abscesses is generally higher (approximately 1/3 of the examined cases, by culture-based detection).^12^^,^^27^ Compared to those using the conventional microbiology methods, studies using molecular diagnostics suggested an overall higher prevalence that varied from 5%^24^ to 65%^7^ (Table 1). The higher prevalence of PBAs was associated with the application of 16S rRNA PCR that detected multiple bacterial species in most cases according to some recent molecular studies.^6^^,^^7^ In the studies using 16S rRNA PCR, the molecularly analyzed samples were the same/residual collections directly from surgery for routine microbiology (Table 1); the detected bacterial species were considered to be pathogenic rather than contaminants, as those studies implemented specific measures such as: (1) clinical correlation,^13^^,^^28^ (2) exclusion of cases with possible contamination,^6^^,^^7^ and/or (3) setting a threshold/frequency with a certain percentage of sequencing read counts to cut-off contaminating sequences with low-frequency reads to prevent false-positives in BA samples.^7^^,^^24^^,^^29^

**Figure 1.**
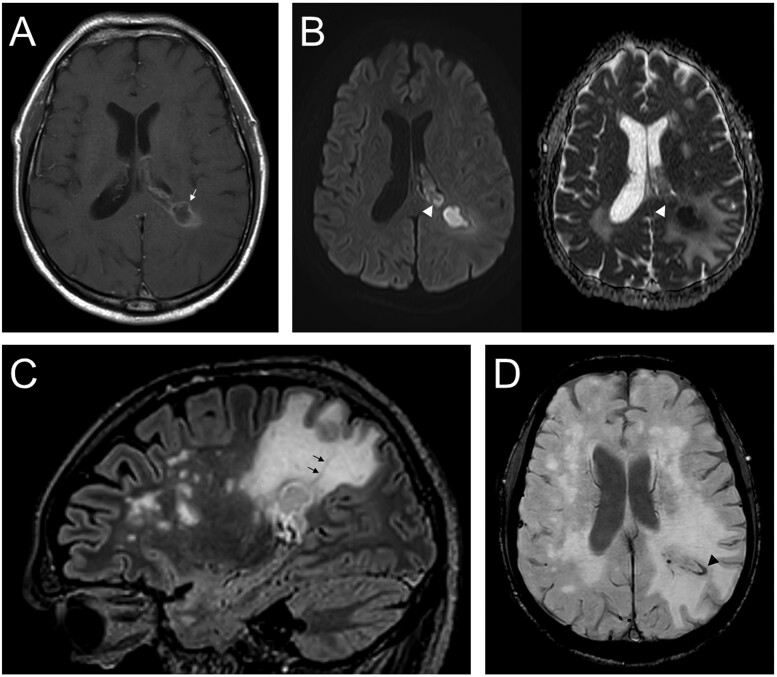
MRI of brain abscess with *Nocardia* and *Toxoplasma* coinfection. (A) Axial T1-weighted image demonstrates a peripherally enhancing left parietal lesion with central T1 hypointense complex fluid contents (arrow). (B) On diffusion-weighted imaging (left) and the corresponding apparent diffusion coefficient map (right), the lesion exhibits central diffusion restriction extending into the ventricle (arrowhead) indicating internal pus and pyogenic ventriculitis. (C) Sagittal FLAIR acquisitions confirm communication between 2 multilobulated components (arrows). (D) There is substantial hypointense signal dropout on susceptibility-weighted imaging, indicating intralesional hemorrhage (arrowhead).

**Figure 2.**
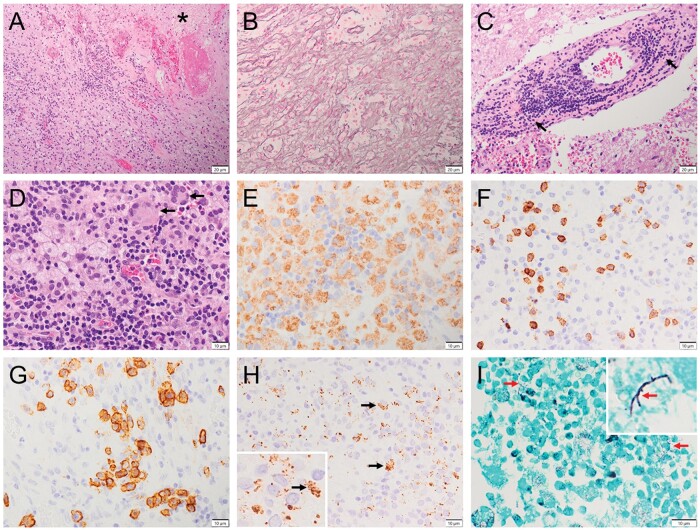
Pathology of concurrent brain *Toxoplasmosis* and *Nocardiosis*. (A, B) Microphotographs of the left parietal lesion show an abscess with necrosis (A, hematoxylin & eosin; *necrotic focus) surrounded by inflammatory cell infiltrates and other reactive changes such as gliosis/fibrosis forming a capsule highlighted by abundant Reticulin staining (B). (C) Nonnecrotic foci exhibit vasculitic changes with transmural inflammatory cell infiltration and some destruction of the vessel wall (arrows, elastic lamina breakdown). (D) Multinucleated giant cells are focally identified (arrows). (E-H) Immunohistochemistry reveals abundant CD68+ macrophages (E) and frequent CD8+ T-cells (F), and CD138+ plasma cells (G), as well as *Toxoplasma*+ tachyzoites (H, arrows; an inset of higher magnification). (I) Grocott-Gomori methenamine silver staining shows focally scattered filamentous branching, beaded bacteria (arrows; an inset of higher magnification). Scale bars: A-C = 20 µm, D-I = 10 µm.

For some nonbacterial pathogens such as *Toxoplasma* and viruses, there is concern about underdetection as these pathogens are not detectable using conventional culture-based methods unless IHC and/or specific PCR is used. There have been a few cases of coinfection of *Toxoplasma* or *Cytomegalovirus* (CMV) with other pathogens (Table S1). Our recent studies have demonstrated 2 cases of coinfection by *Toxoplasma* with *Nocardia* species.^30^^,^^31^ One of these cases was a 63-year-old man who had a culture-confirmed *Nocardia* abscess in the left deltoid muscle; 11 weeks later, he presented with neurological symptoms and subsequent MRI confirming a brain abscess (Figure 1), which contained *Nocardia* species identified by histology and tissue culture, as well as *Toxoplasma* species positive in IHC and PCR of the lesional tissue (Figure 2).^31^ While brain coinfection of *Nocardia* or *Toxoplasma* with other pathogens is seemingly rare (Table S1), this kind of brain coinfection may be underreported, as some brain infections such as nocardiosis and toxoplasmosis can be asymptomatic (even with normal imaging in toxoplasmosis), but become reactivated when another pathogen coinfects the brain.^16^^,^^31^^,^^32^

## Pathogenesis of polymicrobial brain abscesses

The CNS has been recognized as an immunologically specialized microenvironment with several mechanisms to maintain its immunological and pharmacological privileges.^16^ It is separated from the systemic circulation by the blood–brain barrier (BBB) or the blood-cerebrospinal fluid (CSF) barrier, which limits the entry of immune cells and immune mediators into the CNS. In addition, the CNS has other immune features including a lack of traditional connections to the systemic lymphatic system, paucity of dendritic cells in the parenchyma, as well as a delicate neural network sustained by anti-inflammatory factors with local homeostasis. Instead of the systemic lymphatic system, the CNS glymphatic system, which utilizes perivascular channels formed by astroglial cells, serves as a fluid exchange and transport system.^33^ These distinct immune features in the CNS compared to those in non-CNS organs are the basis of CNS immune privilege. Nevertheless, the trafficking of immune cells into the CNS can increase considerably in infection and/or other disease conditions. When the CNS is infected, inflamed, or injured, the infiltration of circulating immune cells and other immune molecules into the CNS has been proposed via 3 mechanisms: (1) through the choroid plexus into CSF that enters the subarachnoid space and circulates around the brain, (2) via the venous sinus to be resorbed by blood enroute to the subarachnoid space, and (3) from blood supply into the perivascular space or Virchow–Robin space and then crossing the disrupted BBB.^16^^,^^34^ The glymphatic function is also impaired in CNS disorders, which could further exacerbate injury due to the accumulation of both metabolic waste and injury-induced debris.^33^ The formation and progression of BAs depends upon direct interplay between the host immune response and virulence of the offending microorganism.^12^^,^^16^

BAs are relatively localized infections, and their formation occurs via contiguous or metastatic spread.^1^^,^^12^^,^^27^ The entry point of microbial infection is informative and predictive of the most likely pathogens involved and thus the selection of optimal empirical antimicrobial therapy.^2^ The common sources of BAs include direct extension from a contiguous cranial infection site or indirect cranial infections arising from the paranasal sinuses, middle ear, and/or teeth following head trauma, dental procedure, or surgery. BA location is often related to the source of infection.^2^^,^^4^ Otogenic abscesses are typically located in the temporal lobe and cerebellum; BAs resulting from sinusitis are primarily present in the frontal or temporal lobes; sphenoid sinus infections extending into the cavernous sinus and middle ear/mastoid sinus can spread into the temporal lobe and cerebellum; mandibular dental infections usually affect the frontal lobe. Although there has been a decrease in the incidence of otogenic or sinus-associated BAs,^2^^,^^35^ the most common source of BAs remains direct or indirect cranial infection arising from paranasal sinuses, middle ear, and/or teeth. In fact, PBAs are particularly common with this cranial infectious source (Table S1).^2^^,^^11^^,^^27^

There have been increasing numbers of BA cases of metastatic spread from a distant extracranial infectious source because of longer life expectancies of immunocompromised patients with conditions such as human immunodeficiency virus (HIV), organ transplantations, chemotherapy for cancers, and steroid use.^2^^,^^10^^,^^12^ In these cases, BAs are typically identified in watershed regions and follow vascular distributions. It is well-known that patients with systemic HIV infection are particularly susceptible to opportunistic CNS infections such as toxoplasma encephalitis, progressive multifocal leukoencephalopathy (PML), cryptococcal meningitis, CMV infection, and tuberculous meningitis.^36^ Many cases of PBAs caused by opportunistic pathogens have been reported in HIV-infected patients^37–39^ and other immunocompromised individuals.^6^^,^^8^^,^^24^ PBAs are also relatively common in patients with hereditary hemorrhagic telangiectasia who commonly present with foci in the lung and/or brain. Despite BAs overall in approximately 1% of patients with hereditary hemorrhagic telangiectasia, PBAs accounted for about one fourth of reported cases and likely resulted from metastatic spread of a systemic infection.^40^

The entry of pathogens causing PBAs into the brain may occur simultaneously or successively. In some cases of PBAs following cranial surgeries, they are caused by metastatic septic foci or infections of the adjacent structures; the causative pathogens particularly bacteria may enter the brain/CNS at the same time as the surgery or days later.^4^^,^^6^^,^^27^^,^^38^^,^^41^ However, in many other cases of brain coinfection with at least 1 nonbacterial pathogen such as *Toxoplasma* or fungi, the brain is infected via a 2-hit process: the first hit occurs earlier as a symptomatic or asymptomatic brain infection and the second hit is superimposed on the initial infection weeks to months later when the host immune response is further suppressed.^16^^,^^38^ Gonzales Zamora reviewed 7 reported cases of brain/CNS coinfection caused by *Cryptococcus* and *Toxoplasma*, which revealed 3 cases of simultaneous infections and 4 other cases of *Toxoplasmosis* that developed after an episode of cryptococcal meningitis with intervals ranging from 2 to 11 months.^38^ One case report described a coinfection of *Streptococcus suis* and *Nocardia asiatica*, in which the CSF next-generation sequencing was positive for *N asiatica* 32 days after the detection of persistent *S suis* when the patient’s condition deteriorated.^42^ As mentioned earlier, some brain infections such as nocardiosis and toxoplasmosis may be asymptomatic and underdetected but can become reactivated when coinfection by another pathogen occurs with increasing suppression of the host immune response.^16^^,^^31^

## Microbiology and histopathology of polymicrobial brain abscesses

PBAs encompass a wide variety of causative microorganisms including bacteria, fungi, parasites, and viruses. In 89 reported PBA cases with details (Table S1), the pathogens involved in coinfections included: *Streptococcus* species (in 40 cases including 5 cases with 2 or 3 species; 45%), *Peptostreptococcus* species (in 23 cases; 26%), *Fusobacterium* species (in 21 cases; 24%), *Staphylococcus* species (in 12 cases; 13%), *Actinomyces* species (in 11 cases including one case with 2 species; 12%), *Bacteroides* species (in 9 cases; 10%), *Haemophilus/hemophilus* species (in 8 cases; 9%), fungi (in 7 cases including one case with 2 species; 8%), *Toxoplasma* species (in 7 cases; 8%), *Prevotella* species (in 6 cases; 7%), *Mycobacterium* species (in 6 cases; 7%), *Nocardia* species (in 5 cases; 6%), *Aggregatibacter* species (in 3 cases; 3%), *Enterococcus* species (in 3 cases including one case with 2 species; 3%), CMV (in 3 cases; 3%), and others (in less than 3 cases) such as *Pseudomonas aeruginosa* (in 2 cases), *Trypanosoma cruzi* (in 1 case), and *John Cunningham virus* (in 1 case). In these individually reported cases of PBAs, S*treptococcus* species are by far the most common, which is consistent with their frequencies described in non-case report studies (Table 1), and are more common in overall (including monomicrobial) BAs.^6^^,^^7^^,^^13^  *Fusobacterium* is also common in the reported PBA cases and overall BAs; in contrast, *Peptostreptococcus* species is the second most commonly detected pathogen in these reported PBA cases but relatively uncommon in overall BAs (including monomicrobial).

While bacteria predominate PBA pathogens, there are also a number of PBAs caused by fungi, parasites, or viruses. Pathogenic fungi commonly contribute to confections of PBAs particularly in immunocompromised individuals.^43^ The vast majority of causative parasites in PBAs are *Toxoplasma* species. The most prevalent viral pathogen in PBA coinfection is CMV, despite HIV systemic infection being more common. Moreover, *Toxoplasma* species and viruses are likely underreported or underdetected as coinfections since they require thorough histological examination and additional diagnostic workups such as IHC or specific PCR, especially in cases with previously asymptomatic toxoplasmosis.

Pathologically, bacterial BAs are known to have a characteristic and sequential manner in their development with 4 stages: (stage I) early cerebritis/encephalitis, (stage II) late cerebritis/encephalitis, (stage III) early capsule stage, and (stage IV) late capsule stage.^3^^,^^31^^,^^44^ Stage I BA differs from stage II BA in the extent of necrosis as stage I BA has minimal to limited necrosis with microvascular damage but stage II BA contains confluent necrosis. The difference between stage III and IV foci is best highlighted by the extent of reticulin staining deposition,^31^ which may correspond to the thickness of an enhancing rim on neuroimaging. While Gram staining may reveal bacteria on histological slides, the detection of Gram-positive or Gram-negative bacteria is best achieved by microbiology techniques. Nonbacterial BAs are histologically somewhat different from bacterial BAs. Fungal BAs often resemble hemorrhagic infarcts and rarely form a thick capsule but may show granulomatous inflammation. Fungi are usually identifiable on histological slides, particularly with special stains such as Periodic acid-Schiff and Grocott methenamine silver. Brain toxoplasmosis (predominant among parasitic PBAs) is characterized by foci of coagulative necrosis surrounded by inflammatory cells and reactive changes such as neovascularization and gliosis, but scanty fibrous encapsulation. Intracellular and extracellular *Toxoplasma* tachyzoites along with cysts containing bradyzoites can be seen with routine hematoxylin and eosin staining but are more readily identified and distinguished from other protozoa by IHC.^16^^,^^31^ Brain viral infection is typically a nonsuppurative encephalitis. CMV brain infection (predominant in viral coinfection) can cause low-grade nonnecrotizing encephalitis with cytomegalic inclusion cells and microglial nodules, or necrotizing encephalitis with large cystic foci resembling infarcts, or ventriculoencephalitis with hemorrhagic necrosis of periventricular brain tissue but no bacterial BA-like encapsulation. Although viral particles may be suspected on histological examination showing nuclear inclusions, the viral detection or confirmation requires IHC or specific PCR.

The complex histopathology of PBAs can be influenced by multiple factors such as the composition, weights (like sequencing read counts in bacterial detection), and virulence of pathogens, particularly the dominant pathogen. This complexity is demonstrable in a few reported cases of coinfections by 2 or more different classes of microorganisms. In one case of CMV and cryptococcal coinfection, full brain postmortem examination revealed multiple bilateral parenchymal cryptococcal abscesses, and CMV micro-abscesses in different sites.^45^ Another polymicrobial case with full brain postmortem examination showed a few sets of coinfected features that included *Toxoplasma* IHC+ abscesses in the corpus callosum, basal ganglia and cerebellar white matter, demyelinating foci in the cerebral white matter with microscopic changes typical of PML, intranuclear papovavirus inclusions in oligodendrocytes, periventricular lesions of subacute encephalitis with large cells immunopositive for CMV and numerous multinucleated giant cells in the demyelinated areas.^37^ These pathological features are consistent with a coinfection of *Toxoplasma*, CMV, and *John Cunningham* virus/polyomavirus/papovavirus causing PML, but they are not typical of a monomicrobial infection or interpretable by any one of those pathogens. Although the reported PBA cases included many with surgical or postmortem pathological examination, detailed histopathological description of PBAs is still scarce (Table S1). Figure 2 demonstrates brain *Nocardia* and *Toxoplasma* coinfection that is *Nocardia*-dominant with a well-formed capsule (Figure 2B; given the patient’s history of prior muscle/skin *Nocardia* abscess) and superimposed *Toxoplasmosis* features such as multinucleated giant cells (Figure 2D).^31^

## Neuroimaging of polymicrobial brain abscesses

BAs including PBAs are well studied by diagnostic imaging. On noncontrast CT, a typical BA, particularly a bacterial late-stage lesion, is characterized by a hypodense center often with an internal complex/heterogeneous appearance, an isodense ring, and a surrounding hypodense zone consistent with edema. On post-contrast CT or MRI images, an early-stage bacterial BA can be enhanced, initially punctate in cerebritis and progressing to ring enhancement in later stages.^1^^,^^2^^,^^12^ Typical BAs on MRI are rounded lesions with central hypointense necrosis surrounded by ring-shaped post-contrast enhancement of the capsule on T1-weighted images, central hyperintensity surrounded by a hypointense capsule on T2/FLAIR images, peripheral vasogenic edema with a T1-hypointense and T2-hyperintense zone, as well as central diffusion restriction on diffusion-weighted imaging. The peripherally or ring-enhancing appearance corresponds to the capsule formation. As described in BA histopathology, the capsulation is only marked in late-stage bacterial BAs; it is poorly formed in non-bacterial BAs and absent in early-stage bacterial BAs or other brain infectious lesions such as viral coinfection. Therefore, early-stage BAs/PBAs are radiologically nonspecific and indistinguishable from their mimics.^12^^,^^31^

In the previously reported cases of PBAs (Table S1), MRI with or without CT characteristics was described in the majority of recent cases while CT features were noted exclusively in earlier cases. PBAs tended to occur more on the left than the right cerebral hemispheres (43 on the left versus 37 on the right, of the side-specified cases), as reported similarly in overall BAs.^27^^,^^46^ This left-sided predominance in PBAs/BAs has been attributed to a greater relative incidence of left-sided penetrating trauma, which is presumably secondary to the predominantly right-handedness of attackers.^47^ The PBA locations (often related to the source of infection, as mentioned previously) included the frontal lobe (most common; in 42 cases, approximately 50% of the reported PBAs), parietal lobe (in 25 cases), temporal lobe (in 16 cases), occipital lobe (in 8 cases), cerebellum (in 10 cases), and brainstem (in 2 cases); multifocal PBAs were identified in 21.3% (19 out of 89) of the reported PBA cases, which is slightly higher than that of overall BAs (7.8%–19.5%).^3^^,^^27^^,^^46^

Imaging features of PBAs may be similarly complex as corresponding to the aforementioned pathological changes; PBAs cannot be diagnosed by imaging alone without microbiology or pathological examination. In the reported PBA cases (Table S1), many lesions dominated by bacterial infection are characteristic, usually with typical imaging features including central necrosis and surrounding ring enhancement with variable thickness. Central restricted diffusion is typically seen, attributed to the restriction of water proton mobility within the cavity due to either necrotic debris and viscous pus or viable bacteria mixed with inflammatory cells.^48^ MRI in a PBA caused by *Actinomyces israelii* and *Fusobacterium nucleatum* showed a mass in the right temporo-parietal-occipital junction with hyper-/isointense-T2 content and significant surrounding edema on T2 images, relatively regular and homogeneous enhancement on T1 post-contrast images, and diffusion restriction in the mass on DWI/ADC images.^49^ A PBA with 4 bacterial pathogens had MRI exhibiting a right temporal cystic lesion with central restricted diffusion, subtle peripheral enhancement, and surrounding vasogenic edema on the DWI, T1 post-contrast, and T2-FLAIR images respectively.^50^ In another PBA with 5 causative bacteria including *Mycobacterium Avium Complex*, CT demonstrated a ring-enhancing, centrally necrotic, bilobed, predominantly left frontal lesion; MRI confirmed features diagnostic of a large intraparenchymal abscess with a complex bilobed appearance, central restricted diffusion, extension involving the left cribriform plate, ethmoids, and posterior table of the frontal sinus.^51^ It is possible that radiologically atypical PBA foci are underreported or less shown.

PBAs with nonbacterial coinfection often show imaging complexity. In a case of brain *Toxoplasma* and CMV coinfection, CT demonstrated extensive low attenuation with mild mass effect in the cerebral white matter bilaterally and left basal ganglia, suspicious for viral infection; subsequent contrast-enhanced MRI revealed multiple bilateral supratentorial and infratentorial ring-enhancing lesions with extensive vasogenic edema and peripheral ring-like low diffusivity (corresponding to the area of enhancement, but without central low diffusivity), which is suspicious for toxoplasmosis.^39^ In Figure 1, an MRI of brain *Nocardia* and *Toxoplasma* coinfection shows a partially deep/centrally located lesion with associated ventriculitis, which is less typical for *Nocardia or* bacterial BA (compared to the other reported cases usually with more peripheral distribution) presumably due to the coinfection with toxoplasmosis that is commonly central and often with ventricular involvement.^31^ Likewise, MRI showed a small volume of intralesional hemorrhage, uncommon for bacterial BA compared to toxoplasmosis.^16^^,^^31^

## Clinical manifestations of polymicrobial brain abscesses

Clinical manifestations of PBAs vary depending on several factors including the host immune response, composition of causative pathogens, virulence of pathogens involved, as well as location and size of lesions. In the reported 89 PBA cases from our literature review (Table S1), the patients (aged from 3 weeks to 80 years) included 17 children; 60 males and 27 females, with a male predilection; 5 individuals had a history/comorbidity of HIV infection; 6 individuals were on immunosuppressive treatment, and 1 patient with cancer had received chemo-radiation therapy. The patients most commonly (60%) presented with headache (51 patients including one with migraine), followed by fever (21 patients; 24%), hemiparesis (20 patients; 23%), vomiting (14 patients; 16%), altered consciousness or mental status (8 patients; 9%), seizure (8 patients; 9%), nausea (7 patients; 8%), and dizziness (6 patients, 7%). While these presentations are largely nonspecific, making the initial clinical diagnosis and management challenging, a thorough history and examination with a low threshold for imaging in patients with prolonged headache and/or other neurological symptoms would aid in identifying the cause and facilitate prompt referrals and management.

In the aforementioned 2-hit process of PBA, the first infection may be asymptomatic; clinically significant infection may not manifest prior to the second hit in the infectious process.^16^^,^^38^ In other words, the non-dominant pathogen(s) may be of little clinical significance but rather an incidental finding on histopathological and/or microbiological studies such as bacterial 16S rRNA sequencing. The incidental finding of a likely nondominant pathogen is exemplified by one case of systemic and brain nocardiosis with coexisting brain toxoplasmosis (Figure 2).^31^ In another reported PBA case, *Mycobacterium tuberculosis* and *Pseudoramibacter alactolyticus* coinfection was detected by the 16S rRNA PCR in a patient with recent dental extraction but no history or evidence of tuberculosis infection; the authors attributed the source of *Mycobacterium tuberculosis* to dental chronic inflammation as oral manifestation represents roughly 1% of tuberculosis cases.^52^ A few studies have suggested that coinfections of bacterial and fungal pathogens are detected in the brains of patients with some neurodegenerative and/or neuroinflammatory diseases such as Alzheimer’s disease and multiple sclerosis, possibly contributing to the pathogenesis and clinical symptoms of those diseases. These coinfections may not give rise to clinical symptoms if they remain below a given threshold, but they can increase throughout the lifetime of an individual and lead to the progressive neurodegenerative symptoms.^53–55^

## Treatment of polymicrobial brain abscesses

BA management requires a prompt multidisciplinary and multimodal approach with both medical and surgical therapies. Empiric broad-spectrum antimicrobial therapy should ideally be commenced when a BA is suspected clinically and confirmed or suggested radiologically. PBAs are not diagnosable until microbiology and/or histopathological findings of 2 or more pathogens in the brain tissue are obtained; therefore, there is no difference in the initial antimicrobial treatment or surgical intervention between polymicrobial and monomicrobial BAs. Prompt neurosurgical management for abscesses that are large, symptomatic, or causing mass effect is paramount in preserving neurological status as well as collecting the lesional tissue for identification of causative pathogens. Depending on the lesion location and radiographical findings, stereotactic aspiration, drainage, or resection of the BA is typically planned unless medically contraindicated.^1^^,^^12^ In our narrative review of 89 PBA cases, 32 patients underwent abscess drainage, 21 underwent abscess resection, 20 underwent needle aspiration, and 8 patients received biopsies. The goal of neurosurgical intervention is 3-fold: (1) histopathological evaluation and PCR to identify causative pathogens as well as guide medical therapy, (2) mass effect reduction, and (3) source control. Multiple interventions are often required.

Initial medical therapy for PBA, before the microbiological or histopathological identification of the pathogens involved, is challenging as the polymicrobial characteristics may be underrecognized and thus undertreated. Empiric broader-spectrum antimicrobial therapy should be initiated in clinically/radiologically diagnosed BA patients with associated paranasal sinus, dental and/or otogenic infections. In HIV-infected or other immunocompromised individuals, anti-toxoplasma/antiparasitic therapy may be considered in correlation with serology testing for *Toxoplasma* antibodies. Once the pathogens are identified, antimicrobial therapy should target the causative pathogens (including the dominant pathogen and those with significant colony counts in PBAs); further management should consider the patient’s predisposing conditions, and the primary source of infection.

## Prognosis of polymicrobial brain abscesses

With the advent of improved diagnosis and treatment of BAs, there has been a substantial decrease in morbidity and mortality during the past decades.^1^^,^^2^^,^^12^ Though historically a fatal disease, BA including the PBA mortality rate declined from 40% in 1960 to 15% after 2000. Higher mortality rates are noted in fungal infections, *Nocardia* infections, brainstem lesions, intraventricular rupture of BAs, and immunocompromised patients. Over the past decade, most BA patients have had good outcomes with no or minimal neurologic sequelae.^1^ Our review of the previously reported 89 PBA cases with follow-up data (Table S1) revealed that 41 patients (46%) had a full recovery and 14 patients (16%) died within 1-6 months of follow-up. This mortality rate of PBAs is similar to that in combined monomicrobial and PBAs.

Significant causes of morbidity associated with BAs include intracranial thrombosis with subsequent stroke, raised intracranial pressure, and seizures. Treatment failure or ineffectiveness in some BA cases is at least partially attributed to the presence and virulence of multiple pathogens, in which the antimicrobial spectrum is not wide enough to eradicate the causative microorganisms in PBAs. Collaboration with the microbiology service and strict adherence to an antibiotic stewardship policy have the potential to reduce morbidity and mortality.

## Conclusion

PBAs are coinfections of bacteria or bacterial and non-bacterial pathogens such as fungi and parasites. PBAs are somewhat similar to monomicrobial BAs, in that both show nonspecific clinical presentations, a male predilection, and left-sided predominance; however, PBAs are more complex in their pathogenic process and pathological/imaging presentations and present their own diagnostic and therapeutic challenges. The diagnostic challenges of PBAs include nonspecific imaging features at early stages and difficulties in the identification of some pathogens by routine microbiology or histological techniques (without PCR). Increasing heterogeneity in these lesions is noted on late-stage imaging and histopathology, particularly in PBA cases with coinfections by nonbacterial pathogens such as fungi or *Toxoplasma* species. PBA therapeutic challenges include the determination of initial medical therapy for possibly underrecognized coinfections prior to identification of multiple pathogens and subsequent broader-spectrum antimicrobial therapy to eradicate the identified pathogens. More awareness of PBAs will facilitate prompt and appropriate treatment of brain infections.

## Supplementary Material

nlae058_Supplementary_Data
