# Supplementary material for: Polymicrobial brain abscesses: A complex condition with diagnostic and therapeutic challenges
Source: J Neuropathol Exp Neurol. 2024 Jun 14;83(10):798–807. doi: 10.1093/jnen/nlae058 (PMC11413443; doi:10.1093/jnen/nlae058)
Supplement: nlae058_Supplementary_Data [file nlae058_supplementary_data.doc]

**SUPPLEMENTAL TABLE 1. Clinical, Radiologic and Pathologic Characteristics of Patients with Polymicrobial Brain Abscesses***

| **Case/Reference** | **Age (yrs), Sex** | **Clinical Presentation** | **Possible Cause** | **PMH** | **Brain Imaging: pre-OR MRI (and/or CT)** | **OR** | **Pathologic Features** | **Pathogens identified in brain tissue (and/or CSF**)** | **Medication Treatment (relevant)** | **F-U Duration/ Outcomes** |
| --- | --- | --- | --- | --- | --- | --- | --- | --- | --- | --- |
| 1. Kisiel et al. 2023 | 63, M | Fever, headache & confusion for 7 wks following muscle/skin abscess | Skin/muscle Nocardia (cultured) abscess,  hematogenous spread | Unremarkable previously; deltoid abscess following COVID-19 vaccine (7 wks prior) | Left parietal multiloculated lesion, 49x21x20 mm, with homogeneous central restricted diffusion, mixed thin/thick peripheral enhancement, & small volume intralesional hemorrhage; associated ventriculitis (Fig. 1) | Resection | Encephalitic changes with necrotic foci surrounded by reactive changes; filamentous branching, beaded bacteria; IHC & PCR *Toxoplasm*a+ (Fig. 2) | *Nocardia sp.,* & *Toxoplasma sp.* | Imipenem/ cilastatin IV; Septra IV, then PO x12 wks | Seizure following discharge, treated, & then, seizure free; 11 mo MRI: post-OR changes; neurologically stable |
| 2. Lamtri Laarif et al. 2023 | Age not specified, M | Frontal headaches, progressively decreased visual acuity for several wks; several episodes of intermittent dysarthria | Dental infection | Unremarkable; immunocompetent | Lesion with hyper-/isointense-T2 fluid content, diffusion-restricting; regular, homogeneously enhanced walls in the right temporo-parietal-  occipital  junction | Drainage | Brain parenchyma with numerous necrotic, abscessed cavities, neutrophils & lymphocytes; rare colonies of *Actinomyc*es sp. in the centre of some cavities | *Actinomyces israelii,* & *Fusobacterium nucleatum* | Ceftriaxone IV, metronidazole & vancomycin IV | Died, during hospitalization; after massive ventriculitis |
| 3. Chen et al. 2022 | 66, M | Dizziness, nausea, vomiting x 4 days |  | No specific disease | Brain abscess |  |  | *(CSF) Nocardia asiatica* & *Streptococcus suis* | Ceftriaxone x14.5 wks, penicillin sodium x14.5 wks, sulfamethoxazole x9 wks, trimethoprim & sulfamethoxazole x52 wks | 12 mos, left hearing loss |
| 4. Wisutep et al. 2022 | 80, F | Progressive, diffuse headache x1 day; sudden onset of aphasia & right-sided weakness; focal clonic movements of right face, upper & lower extremities | Odontogenic | Unremarkable | Left parietal thin rim-enhancing lesion with internal restricted diffusion & surrounding edema | Aspiration | Cytology: polymorphonuclear cells, Gram-positive bacilli | *Porphyromonas gingivalis & Filifactor alocis* | Ceftriaxone IV x2 wks, metronidazole x2 wks, ampicillin (6 wks) & metronidazole x6 wks | 8 mos, full recovery |
| 5. Altdorfer et al. 2021 | 62, sex not specified | Progressive language  impairment, mild dysarthria & neck pain x 5 days; sudden onset of aphasia & confusion | Odontogenic |  | Left parietal mass, 47x43x28 mm, with peripheral enhancement & low apparent diffusion coefficient values | Drainage |  | *Aggregatibacter aphrophilus* & *Actinomyces meyeri* | Cefotaxime x3 mos, vancomycin x4 days & metronidazole x1 mo | 6 mos, full recovery |
| 6. Graham et al. 2021 | 61, F | Generally unwell, confusion & right-sided weakness | Immunocompromised | Hepatitis C, liver transplantation (immunosuppressive therapy), type 2 diabetes mellitus, hypertension, & dyslipidemia | Multiple abscesses in left frontal lobe, up to 23 x10 mm | Resection | Encapsulated foci with necrosis, inflammatory cell infiltrates, & surrounding gliosis/fibrosis; IHC & PCR *Toxoplasma*+; filamentous branching, beaded bacteria | *Nocardia sp.* & *Toxoplasma* | Ceftriaxone x3 wks, trimethoprim/sulfamethoxazole x4 wks, pyrimethamine x1 wk, & leucovorin x1 wk | 3 mos, stable with encephalomalacia |
| 7. Ryu et al. 2021 | 38, M | Severe headaches with painful  neck stiffness, fatigue, myalgia, loss of appetite, & significant  weight loss x1 mo | Dental, suspected |  | Right temporal cystic lesion, 2.5 cm, with peripheral enhancement  & restricted diffusion, extending to the external & internal capsule; additional foci in right temporal lobe, basal ganglia, & internal capsule | Drainage | Fibrinopurulent abscess with necrosis & inflammation | *Fusobacterium nucleatum,*  *Corynebacterium striatum*, *Corynebacterium minutissimum,*  & *Propionibacterium acnes* | Penicillin G IV, metronidazole PO & ceftriaxone IV x6 wks, followed by antibiotics x3 mos | 6 mo MRI: recovery |
| 8. Abo-Zed et al. 2021 | 59, M | Acute onset of left-sided weakness & altered mental status x days | Periodontal disease | Diverticulosis, chronic back pain | Right parietal, peripherally enhancing mass, 2.5 x 2.7 cm, with central marked diffusion restriction, surrounding vasogenic edema & localized mass-effect | Aspiration |  | *Actinomyces, Fusobacterium, Peptostreptococcus*, & *Diphtheroid* | Meropenem & vancomycin IV x6 wks, followed doxycycline PO x6 mos | MRIs x2: resolution of the abscess |
| 9. Takahashi et al. 2020 | 55, F | Blurred vision, eye pain x1 wk | Odontogenic | Unremarkable | Left occipital falsely enhanced lesion, 1.5 cm, surrounded by a low density area; 8 days later, an increase in ring-shaped enhancement with DWI high signal and widening FALIR high signal; 17 days later, ring-enhanced lesion with ventricular puncture suspected | Drainage |  | *Actinomyces cardiffensis* & *Parvimonas micra* | Vancomycin IV x8 days, meropenem IV x22 days, ampicillin IV x74 days, metoronidazole PO x 110 days | 91 days, clinical recovery |
| 10. Rotter et al. 2020 | 60, M | Sinus congestion & headache x2 wks | Sinusitis | Resection of left frontal sinus & zygomaticomaxillary mucocele; Grade 3 colorectal adenocarcinoma, resection & adjuvant chemotherapy | Intraparenchymal, bilobed (5.6x3.1x4.4 cm & 5.7x3.3x3.5 cm) abscess with restricted diffusion & additional  extension involving left cribriform plate, ethmoids, & frontal sinus | Resection |  | *Mycobacterium avium Complex, Achromobacter xylosoxidans, Staphylococcus epidermidis, Streptococcus mitis,* & *Streptococcus salivarius* | Ceftriaxone, ertapenem IV x6 wks, trimethoprim/sulfamethoxazole, levetiracetam PO; meropenem IV, clarithromycin, rifampin, & ethambutol PO x6 mos | Clinical recovery; 1 & 4 mo MRIs: post-OR changes without evidence of residual or recurrent infection |
| 11. Chandra et al. 2020 | 70, M | Right-sided facial weakness, upper & lower extremity numbness x1 day | Paranasal sinus involvement | Unremarkable PMH, except concomitant parasagittal meningioma | Left frontal/parietal enhancing lesion, 2.6x2.1cm, with increased DWI signal and corresponding ADC low signal | Biopsy & drainage | Biopsy tissue confirming meningioma; drainage fluid for microbiological analysis | *Streptococcus constellatus, Fusobacterium sp., Prevotella dentalis,* & *Parvimonas micra* | Levetiracetam, dexamethasone, vancomycin, ceftriaxone, & metronidazole x6 wks | 4 mos, mild residual weakness with observation of meningioma |
| 12. Lo et al. 2020 | 39, M | Convulsions | Immunocompromised | Psoriatic arthritis (on adalimumab) | Left frontal ring-enhancing lesion with perifocal edema. | Drainage |  | *Streptococcus sanguinis, Fusobacterium nucleatum, & Parvimonas micra* | Vancomycin, cefepime, metronidazole, teicoplanin, & ceftriaxone | 4 mo, MRI: “residual granulomatous tissue”; no neurological signs |
| 13. Liao et al. 2019 | 44, M | Fever & progressive headache x2 wks | Dental | Dental extraction (2 days prior); no tuberculosis history | Right temporal enhancing mass with surrounding edema | Drainage | Tissue containing abundant Gram-positive bacteria & acid-fast staining-positive bacteria, with multinucleated giant cells | *(Histology & 16S rRNA sequencing) Mycobacterium tuberculosis* &  *Pseudoramibacter alactolyticus* | Metronidazole & anti-*tuberculosis* treatment (isoniazid,  rifampin x12 mos, pyrazinamide,  ethambutol x2 mos | 12 mo, fully recovered |
| 14. Shafquat et al. 2019 | 10, M | Fever, headache x 2 wks & drowsiness x 2 days | Cardiac predisposition | Tetralogy of Fallot (uncorrected) | (CT) left frontal ring enhancing lesion surrounded by edema | Craniotomy to collect purulent specimen |  | *Aggregatibacter aphrophilus* & *β haemolytic Streptococci Lancefield group F* | Ceftriaxone IV x6 wks | 1 mo, full recovery with no neurological deficits |
| 15. Frenchu et al. 2019 | 63, F | Stupor & facial droop | Cardiac predisposition | Atrial & ventricular septal defects (repaired in her 20s), Afib | (CT) right frontal abscess with vasogenic edema | Aspiration |  | *Mixed oral flora* | Antibiotics, steroids, & seizure prophylaxis | Dizziness |
| 16. Sood et al. 2019 | 40, M | Headache x3 days & altered sensorium x1 day | Immunocompromised | Renal allograft transplantation (6 yrs prior) & pulmonary tuberculosis (2 yrs prior) | Multiple ring-enhancing lesions with moderate perilesional edema in left frontal lobe; the largest: 2.6x1.6 cm | Aspiration |  | *Toxoplasma sp.* & *Cladophialophora bantiana* | Liposomal amphotericin, flucytosine, voriconazole, pyrimethamine, folinic acid, & sulfadiazine | Died, during hospitalization ; repeat CT: massive intracranial hemorrhage |
| 17. Gonzales Zamora et al. 2018 | 52, M | Sore throat & dysphagia to solids & liquids x5 days | Tonsillitis | Unremarkable | Multiple (≥ 28) ill-defined enhancing supratentorial & infratentorial lesions, incl. 8.1x18.1 mm left dorsal midbrain, 10x15 mm left pons, ), 12x17.7 mm left cerebellar, & 7.4x8,6 mm left parietal lesions | Periodic evacuatory lumbar  punctures |  | *(CSF) Toxoplasma sp. & Cryptococcus sp.* | Sulfadiazine, pyrimethamine, leucovorin, liposomal amphotericin, & flucytosine | Died |
| 18. Nkamga et al. 2018 | 30, F | Worsening, persistent headaches x4 days |  | Hereditary haemorrhagic telangiectasia with arteriovenous pulmonary malformation | Left temporal abscess | Aspiration |  | *Aggregatibacter actinomycetemcomitans,* & *M. oralis* |  | 6 mos, clinical and radiological full recovery |
| 19. Chatzaraki et al. 2017 | 48, M | Found dead in prone position | Sinusitis | Frontal sinusitis & erosion of the frontal sinus posterior wall | (Post-mortem CT) left frontal, well-defined, centrally hypodense lesion, 6x3 cm,with a hyperdense ring. |  | Post-mortem confirming brain abscess | *Streptococcus anginosus & Staphylococcus hominis* |  | Died |
| 20. Mashiko et al. 2017 | 72, M | Persistent purulent  discharge from right forehead wound following a trauma 2 mos ago | Trauma causing forehead cellulitis | head trauma 2 mos prior | (CT) right frontal abscess with peripheral enhancement | Resection |  | *Streptococcus salivarius, Streptococcus intermedius, Streptococcus anginosus & Pseudomonas aeruginosa* | Meropenem x6 wks & metronidazole 6 wks | 6 mos, full recovery |
| 21. Srikumar et al.2017 | 67, M | Large scalp wound; left arm clumsiness, altered mental status & low-grade fever | Scalp surgery; immunocompromised | Hypertension, dyslipidemia, myocardial infarction (stent placement), scalp angiosarcoma, treated with chemotherapy, radiation & recurrent/metastatic | Multifocal (2) right frontal ring-enhancing intra-axial lesions with surrounding vasogenic edema | Drainage |  | *Aspergillus terreus* & *gram-positive cocci in clusters* | Dexamethasone x4 wks, cefepime x4 wks, & metronidazole x4 wks, vancomycin, cefepime, dexamethasone, trimethroprim/sulfamethoxazole, & voriconazole x6 mos | 6 mos, loss of fine motor skills & wrist strength |
| 22. Wang et al. 2015 | 42, F | Enlarged pseudofontanelle 15 wks after surgery for a large acute left middle cerebral artery (MCA) stroke | Decompressive hemicraniectomy for a large acute left MCA stroke | Stroke 15 wks prior | Rim-enhancing collection in the previous infarcted area of left MCA territory, suspicious for tissue necrosis with infection forming an abscess | Resection of infarcted brain tissue |  | *Pantoea agglomerans & Bacillus macerans* | Vancomycin x6 wks, cefepime x6 wks, & flagyl x6 wks | 16 mos, full recovery |
| 23. Greninger et al. 2015 | 40, F | Right frontal headache x7 days; night sweats, chills, vomiting, left-sided vision loss, & right leg numbness x2 days |  | Migraines, & frequent childhood sinopulmonary infections | Right parietal rim-enhancing lesion, 3.0x 3.7x2.8 cm, with restricted diffusion | Drainage |  | *Streptococcus anginosus, Peptostreptococcus sp.,* & *Mycobacterium immunogenum* | Vancomycin, metronidazole, & ceftriaxone | 5 wks, a stable visual field defect, resolved headache and sensory  Changes; imaging: a collapsed abscess cavity |
| 24. Bogdan et al. 2015 | 13, M | Headache, dizziness & vomiting | Odontogenic | Healthy | Right parietal solitary expansive mass, 41x31 mm, with peripheral enhancement & peripheral edema | Resection (complete evacuation of purulent material and capsule) |  | *Hemophilus aphrophilus* & *Bacteroides uniformis* | Metronidazole, ceftriaxone & cefixime | 15 mos, full recovery |
| 25. Su et al. 2014 | 18, M | Headache, altered consciousness | Trauma | Head injury, skull base fracture | Right frontal lesion, 3 cm | Resection/excision |  | *Streptococcus viridans & Peptostreptococcus sp.* |  | 36 mos, full recovery |
| 26. Jehangir et al. 2014 | 52, M | Found sitting in own excretions, non-responsive at admission | Immunocompromised | Hypertension & alcohol abuse; newly found serology HIV positive, with CD4<13. | Multiple (bilateral supratentorial & infratentorial) ring-enhancing lesions with peripheral ring-like low diffusivity & no central low diffusivity; the largest: 3.4 x 1.9 cm in left frontal lobe |  |  | *(CSF) Toxoplasma sp. &* CMV | Vancomycin, Zosyn, folic acid, thiamine, & acyclovir | Alive |
| 27. Pallesen et al. 2014 | 55, M | Acute onset weakness of left leg | Odontogenic (tooth cleaning 10 days prior) | Unremarkable | Multiple (6) ringlike enhancing lesions with hyperintense signals in DWI & surrounding edema | Resection of 2 lesions |  | *Streptococcus intermedius & Staphylococcus warneri* | Ceftriaxone, metronidazole, vancomycin, & gentamicin | 6 mos, moderate left leg paresis |
| 28. Sacks et al. 2013 | 20, M | Facial trauma and quadriplegia following a self-inflicted gunshot to left forehead | Ballistic  head injury |  | Multiloculated cerebral abscess involving right gyrus  rectus, left gyrus rectus, and left medial orbitofrontal cortex,  with surrounding edema & dural enhancement | Drainage |  | *Coagulase-negative Staphylococcus sp., Diphtheroids sp., & an unclassified yeast* | Vancomycin, meropenem, & micafungin | 12 mos, neurological recovery; CT: well-healed |
| 29. Haane et al. 2013 | 41, M | Seizure with unconsciousness x10 min | Immunocompromised | Crohn's disease x25 yrs, treatment incl. steroids & squamous cell carcinoma | Left temporal abscess | Aspiration |  | *Streptococcus intermedius & anaerobic bacteria* |  | Died |
| 30. Larbcharoensub et al. 2013 | 49, M | Fever, intense headache & right-sided hemiparesis, after  pulmonary aspergillosis 6 wks ago | Systemic infection; immunocompromised | Chronic glomerulonephritis, with cadaveric rental transplantation 14 yrs ago, followed by immunosuppression incl. prednisolone. | Multiple ring-enhancing abscesses,  involving the centrum semiovale, left corona radiata, right occipital lobe & left splenium of the corpus callosum, with perilesional vasogenic edema | Drainage | Multiple fungal elements with dichotomous branching septate hyphae  & irregular, septate hyphal elements with beaded yeast-like forms. | *Scedosporium apiospermum* & *Phaeoacremonium parasiticum* | Voriconazole IV, then PO x6 mos | 72 mos, clinically and radiologically improved; no recurrence |
| 31. Johnson et al. 2013 | 11, F | Headache & right-sided weakness; fever, chills, abdominal pain, & sore  throat x8 days | Odontogenic | Vasovagal syncope; otherwise, unremarkable | Multiple (2) lesions, in left frontal pole (14x13x12 mm) & left superior frontal gyrus (19x18x15 mm), with peripheral enhancement, surrounding vasogenic edema, & restricted diffusion | Biopsy/drainage |  | *Eikenella species* & *Streptococcus milleri* | Antibiotic therapy x9 wks | 9 wks, clinical recovery: MRI: continued improvement |
| 32. Simsek et al. 2013 | 13, F | Headache, vomiting, impairment in gait & stance, long lasting | Immunocompromised | Multiple cutaneous abscesses in infancy, treated for bronchopneumonia. Histiocytosis X diagnosed at one yr of age | Multiple lobulated lesions with nodular or ring enhancement,  cystic & necrotic components in posterior fossa involving both cerebellar hemispheres; the number of lesions doubled in a mo. | Resection/drainage | Caseified granulomatous inflammation with inflammatory cells incl. Langhans giant cells & fungi (branching & budding septate hyphae) | *Paecilomyces variotii* & *Mycobacterium tuberculosis* | Anti-tuberculous therapy (isoniazid, rifampicin, pyrazinamide and streptomycin), started before OR or MRI finding of abscesses | Died, 2 wks after OR |
| 33. Azenha et al. 2012 | 70, M | Headache, dizziness, nausea, fever, left-sided muscle weakness x days | Odontogenic | Unremarkable | Multiple (3) lesions, in in frontal lobe, central brain area, & occipital lobe, with low density on CT and restricted diffusion on MRI/DWI | Drainage |  | *Streptococcus viridans & Bacteroides sp.* | Ceftriaxone, amoxicillin, & metronidazole | 12 mos, clinical full recovery; CT: post-OR changes |
| 34. Antunes et al. 2011 | 56, M | Migraines, nausea, & convulsions | Odontogenic | Dental infection | Left temporal lesion with hypointense T1, hyperintense T2, & isointense FLAIR with temporal bone destruction | Drainage |  | *Streptococcus viridans, Actinobacillus actinomycetemcomitans, & Staphylococcus sp.* |  | Died, 2 days post-OR |
| 35. Hsu et al. 2011 | 54, F | Altered consciousness, dizziness x20 days | Ear infection | Diabetes mellitus, mastoiditis | Right cerebellar abscess | Excision |  | *Peptostreptococcus micros, Enterococcus faecalis, Enterococcus casseliflavus, Fusobacterium spp.,* & *Shewanella putrefaciens* | Vancomycin x2 wks, ceftazidime x2 wks, metronidazole x4.5 wks, ceftriaxone x4.5 wks, ampicillin x5.5 wks, & ceftazidime x5.5 wks | Poor prognosis with ataxia |
| 36. Hsu et al. 2011 | 51, M | Headache, altered consciousness, dizziness, ataxia, dysarthria x10 days |  |  | Multiple abscesses, right cerebellar | Excision |  | *Peptostreptococcus micros, Viridans streptococcus,* & *Pseudomonas aeruginosa* | Penicillin G x 5.5 wks, gentamycin x5.5 wks, chloramphenicol x5.5 wks, & clindamycin x13 wks | Full recovery |
| 37. Stein et al. 2011 | 47, F | Headache & neck pain x3 days; bronchitis x15 days | systemic infection | Phaeochromocytoma (surgery 7 yrs prior) | Left brainstem/midbrain encapsulated, ring-enhancing lesion | Aspiration |  | *Haemophilus influenza* & *Peptostreptococcus sp.* | Ceftriaxone, ampicillin & metronidazole | 6 mos, moderate deficit, mobilizing in a wheelchair |
| 38. Akhaddar et al. 2010 | 19, M | Episodic  generalized seizure x3 mos; right hemiparesis |  | Open head injury 8 yrs prior, treated with left parietal craniectomy debridement, with a good outcome | Regular cystic lesion with a thin rim of enhancement in the left parietal lobe | Drainage |  | Gram-negative rods, gram-positive rods,  *Pseudomonas aeruginosa & Actino-*  *myces sp.* | Ceftriaxone, gentamicin, &  metronidazole x10 days, oral ciprofloxacin x2 mos | Remains well |
| 39. Diaz et al. 2010 | 48, F | Obtunded with GCS of 8 after a motor vehicle accident | Trauma; immunocompromised | Motor vehicle accident (multiple injuries); hereditary hemorrhagic telangiectasia (multiple, recurrent, or incompletely-embolized  arteriovenous malformations & retained  coils throughout both lungs on CT); HIV infection | (CT) left frontal ring-enhancing lesion | Drainage |  | *Staphylococcus aureus, Streptococcus intermedius, & yeast* | Ceftriaxone, vancomycin, & fluconazole | 4 wks, asymptomatic |
| 40. Keller et al. 2010 | 29, M | First seizure, frontal headache | Sinusitis | acute frontal sinusitis diagnosed 3 days prior | (CT) pansinusitis with a bone defect of the dorsal wall of frontal sinus & adjacent brain abscess | Excision |  | *Fusobacterium nucleatum* & *Porphyromonas endodontalis* | Ceftriaxone x6 wks & metronidazole x6 wks) | 6 wks, full recovery except residual headache |
| 41. Keiner et al. 2009 | 38, M | Brain abscess recurrence | Odontogenic; brain surgeries (10 & 1 yrs prior) | A right frontal cyst treated by endoscopic cystoventriculostomy & cyst biopsy, 10 yrs prior; right frontal abscess, treated by small osteoplastic craniotomy and abscess drainage, 1 yr prior | Right frontal abscess with contrast-enhancing membrane compressing the cyst | Drainage & complete cyst capsule resection | Scar-tissue, necrosis, pus & mucosal tissue | *Enterobacter cloacae, Prevotella denticola, Fusobacterium sulci & Peptostreptococcus tetradius* | Antibiotics | Full recovery without any neurological deficit |
| 42. Sakamoto et al. 2009 | 61, M | Headache x1 wk | Odontogenic | Dental infection, 2 wks prior | (CT) left temporal abscess & parapharyngeal abscess | Drainage |  | *Streptococcus sp., Peptostreptococcus sp., Prevotella sp., Fusobacterium sp.,* & *anaerobic gram-positive bacteria* | Panipenem & betamipron | 63 days, discharged with gradual improvement but disorientation |
| 43. Shachor-Meyouhas et al. 2009 | 2.5, M | Headache, vomiting & facial nerve paralysis x10 days | Systemic infection | Septic arthritis of left knee (culture-negative at age 6 mos) | (CT) right temporoparietal lesion with peripheral enhancement and surrounding edema | Drainage |  | *Fusobacterium sp. & Streptococcus intermedius* | Ceftriaxone x6 wks & metronidazole x6 wks | 6 mo, full recovery |
| 44. Ozüm et al. 2008 | 41, M | Headache, vomiting & right-sided weakness | Odontogenic | Left upper tooth pus with drainage 6 mos prior | Right parietal ring-shaped enhancing lesion with perifocal edema | Drainage | Abscess material showing few inflammatory cells & few  trophozoites | *Eikenella corrodens* & *Prevotella sp.* | Penicillin-G, metronidazole & anti-oedema agents | 6 wks, discharged |
| 45. Pham et al. 2008 | 36, M | Right extremity weakness, mild function control, & mild convulsion | Trauma | Head trauma (motorbike accident) with minor cerebral thrombosis | Right parieto-occipital tumor-like lesion, suspicious for cancer metastasis | Resection of well-delineated, circumscribed lesion | Central necrosis & granulomatous tissue in the periphery &  fungus hyphe. | *Candida albicans* & *Cladophialophora bantiana* | Fluconazole IV x2 wks | Elected to withdraw care, after MRI showing increased mass size |
| 46. Roberts et al.2008 | 10, M | Headache, nausea, dizziness, & weight loss x2 wks; vomiting & intermittent diplopia x3 days | Systemic infection | Pushpin in left main stem bronchus, removed by surgery | Right front large, well-encapsulated abscess | Resection of the abscess & entire thick fibrous capsule |  | *Actinomyces meyeri, Actinomyces sp. & Haemophilus aphrophilus* | Vancomycin,  cefotaxime & metronidazole, followed by G  monotherapy. | 21 days, discharged fully recovered |
| 47. Ramesh et al. 2008 | 24, M | Headache, neck pain & gait unsteadiness x2 mos | Systemic infection | Pulmonary tuberculosis, diagnosed 6 mos ago | Large ring-enhancing lesion in cerebellar vermis & right hemisphere with obstructive  hydrocephalus | Resection/excision of the abscess thick capsule | Fibrocollagenous tissue with inflammatory cells & no evidence of Langhan's giant cells or caseation | *Mycobacteria sp.* & *Staphylococcus aureus* | Antibiotics & antituberculous chemotherapy | Uneventful recovery |
| 48. Yilmaz et al. 2007 | 28, M | Fever, frontal headache & left ear pain x4 days | Ear infection; mastoiditis | chronic suppurative otitis media since childhood | Cerebellar abscess, 3x1 cm, with cerebellar edema & enhancement of lateral sinus & mastoiditis | Drainage |  | *Shewanella putrefaciens* & *Klebsiella pneumoniae* | Meropenem | 8 wks, recovered; discharged without any neurological deficit |
| 49. Kombogiorgas et al. 2007 | 13, F | Hemiparesis, learning  difficulties | Ear infection | Mastoiditis | Intracerebral lesion | Aspiration |  | *Proteus oralis* & *mixed anaerobes* | Ceftriaxone x21 days & metronidazole x70 days | Hemiparesis & learning difficulties |
| 50. Young et al. 2005 | 38, M | Bifrontal headache, right ear discomfort, & fever x1 wk | Odontogenic | Unremarkable | Right temporal 2-cm enhancing lesion with edema | Resection of a meningioma & intratumoral & peritumoral abscess | Meningothelial cells with calcifications & acute inflammatory cells | *β Streptococcus* & *Peptostreptococcus sp.* | Oxacillin IV x3 mos | 3 mos, full recovery |
| 51. Strojnik & Roskar 2004 | 12, M | Right hemiparesis | Odontogenic | Milk tooth self-extraction | Left precentral gyrus abscess | Aspiration |  | *Streptococcus intermedius, Streptococcus beta-haemolyticus group F, Fusobacterium species,* & *gram-negative rods* | Antibiotics | Full recovery |
| 52. Friedlander et al. 2003 | 58, F | Left hemiparesis; right frontal headache with radiation to right eye & ear 5 days | Sinusitis | Headaches without radiation | Right frontal heterogeneous mass with central hyperintensity on T2, & central hypointensity surrounded by ring enhancement on T1, hyperintense on DWI & hypointensity on ADC | Aspiration |  | *Streptococcus milleri & Haemophilus aphrophilus* | Antibiotics | Full recovery |
| 53. Rao et al. 2002 | 3 wks, M | Fever, decreased oral intake, irritability, seizure |  | Unremarkable (full term, uncomplicated pregnancy of the mother) | (CT) right frontal ring-enhancing mass, 4x5 cm; 2 wks later, new parietal abscess with increasing dilatation of the ventricular system | Aspiration |  | *Haemophilus influenzae type B, Streptococcus pneumoniae, Neisseria meningitidis,* & *Mycoplasma hominis* | Vancomycin, cefotaxime & metronidazole | Moderate developmental delay; CT showing resolution of the abscesses & decrease of hydrocephalus |
| 54. Cihangiroglu et al. 2001 | 25, F | Right sixth nerve palsy x1 mo | Sinusitis | Pregnancy (25^th^ wk) | Multiple (at least 2) intraparenchymal ring-enhancing lesions in right temporal & parietal lobes, with diffuse edema & diffuse leptomeningeal enhancement | Decompressive craniotomy/resection |  | *Aerobic & anaeorobic microorganisms incl. Peptostreptococcus sp* |  |  |
| 55. Corson et al. 2001 | 56, M | Epileptic fits, right hemiparesis | Odontogenic |  | Left fronto-parietal solitary large irregular lesion with surrounding white matter edema | Resection/excision of the lesion (vascular ill-defined pus filled cavity) | Confirming brain abscess with Gram+ cocci | *Streptococcus milleri* & *Streptococcus sanguis* | Cefotaxime, amoxycillin | 5 mos, completely recovered from hemiplegia, but slightly sub-optimal thinking, concentration & speaking |
| 56. Yamamoto et al. 2000 | 43, M | GCS 15 | Systemic infection | Pneumonia | Multiple (3) abscesses in frontal (5 cm), parietal (3.8 cm), & occipital (1.7 cm) lobes | Aspirations |  | *Fusobacterium necrophorum & Peptostreptococcus micros* | Pipracillin sodium, minocycline hydrochloride, flomoxef, & tobramycin | Full recovery |
| 57. Yamamoto et al. 2000 | 8, F | GCS 14 |  | Hereditary hemorrhagic telangiectasia | Frontal abscess (5.8 cm) | Aspiration |  | *Streptococcus anginosus, Bacteroides fragilis,* & *Fusobacterium necrophorum* | Ampicillin, cefotaxime, & chloramphenicol | Moderately disabled (functional impairment) |
| 58. Yamamoto et al. 2000 | 24, M | GCS 15 |  |  | Frontal abscess (3.8 cm) | Aspiration |  | *Fusobacterium necrophorum & Peptostreptococcus micros* | Ampicillin, sulbactam, & cefoperazone | Full recovery |
| 59. Lozniewski et al. 1999 | 39, M | Maxillary sinusitis, headache, nuchal rigidity, & fever x2 days | Odontogenic | Alcoholic | (CT) left  frontal & parietal large collection with major mass effect & left-sided pansinusitis | Drainages |  | *Streptococcus constellatus, Capnocytophaga sp., Eubacterium*  *exiguum,* & *Desulfovibrio sp.* | Cefotaxime, fosfomycin & ornidazole;  then piperacillin, pefloxacin, & ornidazole |  |
| 60. Koot et al. 1999 | 6 wks, F | Right parietal scalp laceration at birth followed by enlarged head circumference | Trauma | Scalp laceration | (CT) right hemispheric ring-enhanced lesion, 7x 4x3 cm, with surrounding edema & midline displacement | Drainage |  | *Staphylococcus aureus, Bacteroides oralis,* & *Peptostreptococcus sp.* | Penicillin, metronidazole, & floxacillin | 36 mos, hemiparesis; 6 wks, CT showing progressive reduction of the abscess |
| 61. Sahjpaul et al. 1999 | 26, M | Right-sided headache x4 days | Sinusitis | Sinusitis & polypectomy several yrs prior | Enhancing lesions in right cerebellar pontine angle & cerebellar hemisphere posterior fossa abscess, along with diffuse pansinusitis | Resection |  | *Hemolytic Streptococcus* & *coagulase-negative Staphylococcus sp.* | Ceftriaxone IV x3 wks | 1 mo, blind right eye & anterior pituitary failure; MRI showing significant improvement |
| 62. Ruebenacker et al. 1999 | 27, F | Headaches & photophobia x1 mo | Odontogenic | Unremarkable | (CT) left frontal ring-enhancing lesion with a fluid level & gas formation within the lesion & mass effect, along with sinusitis of frontal & maxillary air sinuses | Resection/dissection |  | *Prevotella buccae, Streptococcus millerei, Viridans streptococci, Wolinella sp.,* & *Bacteroides thetaiotamicron* | Metronidazole, ceftriaxone nafcillin, penicillin, phenytoin, & carbamazepine | 30 mos, neurologically intact; full recovery; CT showing resolution of the abscess, despite a seizure at 12 mos |
| 63. Barlas et al. 1999 | 15, M |  | Contiguous  infection | Nasopharyngeal infection | (CT) multiple abscesses (up to 2.5 cm) in cortical & deep white matter | Drainage |  | *Peptostreptococcus sp.* & *Viridans streptococci* |  | 16 mos, recovery |
| 64. Akimura et al. 1998 | 38, M | Left temporal headache & mild fever x48 hours | Contiguous  infection | Recurrent orbital cellulitis | (CT) left frontal rim-enhancing lesion, with acute hydrocephalus & rupture into the lateral ventricle | Drainage with ventriculostomy | Autopsy revealing dural fistula with erosion of orbital plate of the frontal bone  & recurrent orbital cellulitis | *Streptococcus constellatus, Peptostreptococcus sp., Prevotella intermedia,* & *Gemella morbillorum* | Cephalosporin, penicillin & carbapenems | 10 days, died |
| 65. Maniglia et al. 1997 | 41, M | Headache, right arm stiffening (with head turning to the right) | Odontogenic; immunocompromised | HIV x7 yrs & a decayed tooth | (CT) fronto-parietal multiloculated ring-enhancing lesion with white matter vasogenic edema | Biopsy |  | *Streptococcus bovis, Fusobacterium necrophorum, Peptostreptococcus sp.,* & *group C Streptococcus* | Ceftriaxone, vancomycin, & metronida | 42 mos, near-total recovery |
| 66. Tekkök et al. 1996 | 20 mo, F | Imbalance x 3-4 days, followed by nausea, vomiting,  & irritability |  |  | (CT) lobulated, cystic space-occupying lesion in the posterior fossa midline to left cerebellar hemisphere. | Drainage |  | *Corynebacterium aquaticum, Enterobacter sakazakii* & *Enterobacter cloacae* | Ceftriaxone, penicillin & gentamicin IV x6 wks | 30 mos, neurologically intact/recovered & developing well |
| 67. Simpson et al. 1996 | 66, F | Headache, drowsiness & blurred vision x10 days | Odontogenic | toothache | (CT) right occipital irregular mass | Resection/excision of the abscess & capsule |  | *Haemophilus paraphrophilus, Peptostreptococcus micros, Fusobacterium nucleatum, Actinomyces odontolyticus,* & *another unidentified anaerobic gram-negative rod* | Benzylpenicilin, flucloxacilin, gentamicin & metronidazole | Remaining well; visual fields improved |
| 68. Rhatigan & Taylor 1996 | 74, F | Accident fall with right eyelid injury | Trauma |  | (CT) foreign body  passing along the superior orbit & through orbital roof into right frontal lobe of the brain | Foreign body removal |  | *Bacillus circulans & Bacillus laterosporus* | Antibiotics | Full recovery |
| 69. Dethy et al. 1995 | 46, M | Aphasia & diffuse headache | Cardiac predisposition | Silent atrial septal defect | Left Rolandic (fronto-parietal) lesion with peripheral  ring-like enhancement | Biopsy | Brain abscess | *Fusobacterium sp. & Peptostreptoccocus anaerobius* | metronidazole & ampicillin | 12 mos, full recovery |
| 70. Hall 1994 | 26, M | Fatigue & right arm weakness x1 wk; seizure |  | Pulmonary arteriovenous fistulae (in both lungs) & frequent dyspnea on exertion with recurrent epistaxis since childhood. | Left frontal enhancing mass with surrounding edema | Aspiration |  | *Streptococcus intermedius, Actinomyces meyeri, Fusobacterium nucleatum, Capnocytophaga species,* & *Staphylococcus epidermidis* | Chloramphenicol & penicillin G x6 wks | 24 mos, full recovery |
| 71. Navas et al. 1994 | 45, M | Headache & left hemiparesis x5 days |  | Alcohol abuse | Right parieto-occipital ring-enhancing lesion with air bubbles & surrounding edema | Excision/resection | Clumps of Gram+ non-acid-fast, branching filamentous organisms | *Actinomyces israelii* & *Fusobacterium sp.* | Cefotaxime x4 wks, chloramphenicol x4 wks, & penicillin x52 wks | 18 mos, full recovery |
| 72. doi: 10.1056/NEJM199310283291809. 1993 | 71, F | Midbrain frontal headache x3 wks |  | Hypertension, angina pectoris, & subendocardial infarct; seropositive rheumatoid arthritis | Left occipital ring-enhancing lesion, 4.3x 3.7x3 cm, extended into parieto-occipital region; T2 hypointense regions in basal ganglia & periventricular areas | Biopsy |  | *Fusobacterium nucleatum & Peptostreptococcus micros* | Penicillin & metronidazole | 6 mos, mild quadrantanopia |
| 73. doi: 10.1056/NEJM199004193221608. 1990 | 57, F | Left-sided headache, bilateral periorbital pain; fatigue, fall toward the right x3 days | Sinusitis | Hereditary hemorrhagic telangiectasia in both lungs, chronic sinusitis | Left thalamic ring-enhancing lesion with mass effect | Biopsy |  | *Fusobacterium nucleatum, Peptostreptococcus micros, Eubacterium sp., Bacteroides gracilis,* & *Coccobacilliform sp.* | Cefotaxime, ampicillin, & metronidazole |  |
| 74. Gray et al. 1987 | 29, M | Fever, headaches, intellectual slowing, gait disturbance & incontinence | Surgical infection; immunocompromised | HIV/AIDS & nasal operation |  |  | (Post-mortem) IHC *Toxoplasma+* abscesses in corpus  callosum, basal ganglia & cerebellar white  matter; demyelinating foci in cerebral white matter, with intranuclear  papovavirus inclusions in oligodendrocytes, & multinucleated giant cells in demyelinated areas; periventricular foci of subacute encephalitis with IHC CMV+ large cells | *Toxoplasma sp., JC virus/PML, CMV* |  | 4 mos, died |
| 75. Bishburg et al. 1986 | 33, M | Fever, altered mental capacity & right facial weakness 3 mos after disseminated tuberculosis treatment | Systemic tuberculosis | Disseminated tuberculosis | Multiple (x2) ring-enhancing mass lesions in right frontal & left temporoparietal lobes |  | (Post-mortem) large caseous necrotic masses, 3 & 4 cm; containing *Toxoplasma gondii* & *acid-fast bacilli* | *Toxoplasma sp.* & *Mycobacterium tuberculosis* | Isoniazid, rifampin, pyrazinamide, sulfamethoxazoletrimethroprim, & pyrimethamine | Died |
| 76. Chee et al.1986 | 58, M | Giddiness, headache & vomiting x2 days | Ear infection | Purulent discharge from his right ear x 6 mos | (CT) right temporal multi occulated thick-walled abscess | Aspiration |  | *Streptococcus viridans* & diphtheroids |  | 6 mos, full recovery |
| 77. Stallworth et al. 1985 | 27 mo, M | Fever, meningismus & left eye proptosis | Trauma | Left eyelid injury 3 days ago with mild laceration | (CT) left frontal ring-enhancing abscess | Aspiration |  | *Enterococus sp.* & *Nocardia sp.* | Methoxazole x2 wks & trimethoprimsulfamethoxazole x54 wks | 12 mos, full recovery; CT showing complete resolution of the abscess |
| 78. Dijkmans et al. 1984 | 28, F | Headache, fever & muscle tenderness | Contact with rodent or its excreta | Heroin smoking | (CT) multiple lesions incl. circular enhancing lesion in left basal ganglia & mass in left parietal lobe | Aspiration of left parietal lobe & external drainage of left lateral ventricle |  | *Streptobacillus moniliformis* & *Actinobacterium meyerii* |  | 12 mos, full recovery |
| 79. Young & Frazee. 1984 | 13, F | Left ear pain, headache, nausea and vomiting | Ear infection | Chronic mastoiditis (left radical mastoidectomy) | (Roentgenograms) left temporal, large gas collection | Drainage (first) & excision (second) |  | *Klebsiella sp.* & *Staphylococcus aureus* | Ampicillin, clindamycin, & gentamicin | 3 mos, died |
| 80. Young & Frazee. 1984 | 39, F | Headache, vomiting, nuchal rigidity, right hemiparesis, & dysphasia | Ear infection | Chronic otitis media (left radical mastoidectomy) | (CT) left temporal large gas-containing mass | Excision of mass with well-defined capsule |  | *Streptococcus uiridans, Staphylococcus epidemidts,* & *gram negative anaerobic coccobacillus* | Antibiotics | 54 mos, mild deficits of recent memory |
| 81. Young & Frazee. 1984 | 11, F | Pain, swelling, & proptosis of the right eye | Upper respiratory tract infection |  | (CT) right frontal lesion containing gas | Aspiration |  | *Lactobacillus catenafome, Eubacterium lentum, Peptostreptococcus micros,* & *Bacteroides corrodens* |  | 18 mos, normal neurological functions |
| 82. Press et al. 1984 | 67, F | Fever one mo after surgical drainage of salivary gland abscess | Odontogenic | Right submandibular abscess | (CT) large right temporoparietal enhancing lesion with associated edema | Aspiration |  | *Gram positive cocci* & *rods* | Tobramycin & Cefotaxime | 1.5 mos, died |
| 83. Levy et al. 1984 | 37, M | Headache & lethargy | Systemic infection; immunocompromised | Pneumocystis carinii pneumonia, AIDS, CMV, gonorrhea, syphilis, herpes genitalis, amebiasis, giardiasis, moniliasis, hepatitis A & B |  |  | (Post-mortem) generalized meningitis & ventriculitis; multiple bilateral parenchymal cryptococcal abscesses, as well as parenchymal CMV microabscesses in different sites. | *Cryptococcal sp.* & *CMV* | Antibiotics | Died |
| 84. Grace & Drake-Lee. 1984 | 20, M | Right ethmoiditis associated with proptosis & periorbital celulitis | Sinusitis | Left frontal sinusitis | (nspecified imaging) right frontal abscess | Resection (partial) |  | *Streptococcus milleri* Lancefield group *C, Haemophilus aphrophilus,* & *Bacteroides melaninogenicus* | Metronidazole, enzylpenicillin & chloramphenicol | Full recovery |
| 85. Sable et al. 1984 | 12, M | Headache, nasal stiffness, vomiting & low grade fever x6 days | Sinusitis |  | (CT) left frontal cerebritis, left frontal sinusitis & anterior ethmoiditis | Drainage |  | *Streptococcus sanguinis* & *Peptostreptoccocus sp.* | Metronidazole & ampicillin | Discharged with residual diplopia & decreased memory |
| 86. Härkönen 1981 | 21, M | Headache & fever | Sinusitis | Hereditary Hemorrhagic Telangiectasia with pulmonary arteriovenous fistula | (CT) multiple (2) thick-walled abscesses in right temporal lobe | OR |  | alpha-hemolytic *Streptococci sp.* & *enterococci sp.* |  | 12 mos, alive with epilepsy |
| 87. Warner et al. 1979 | 25, M | Severe headache; transferred for treatment of a persistent right frontal brain abscess x3 mos |  |  | (CT) abscess/cerebritis occupying most of right frontal lobe | Drainages |  | β hemolytic *Streptococcus, Bacteroides sp., Fusobacterium naviforme,* & *Peptostreptococcus sp.* | Penicillin G, ampicillin, chloramphenicol, & metronidazole | Full recovery |
| 88. Román et al. 1978 | 72, F | Nausea, vomiting, headache, fever, & epistaxis |  | Hereditary hemorrhagic telangiectasia, with OR for left lung arteriovenous fistula; transient ischemic attack | (CT not available) cerebral angiogram showing multiple arterial malformations |  | (Post-mortem) large abscess in the vermis extending into right & left cerebellar hemispheres; telangiectasia foci in multiple brain regions | *Nocardia spp.,* Gram+ cocci & rods |  | 1 mo, died |
| 89. Adams et al. 1977 | 29, M | Headache & fever x1 wk; vomiting, confusion, & progressive stupor | Vascular malformations with OR | Hereditary hemorrhagic telangiectasia; multiple small vascular malformations in both hemispheres & right frontal avascular mass (hematoma at OR) | (CT) left frontal large mass with decreased density in the center surrounded by vascular ring & edema;  angiograms showing middle cerebral artery vascular malformations | Drainage |  | *Peptostreptococcus sp.* & *Hemophilus aphrophilus* |  | Improved initially; 2 mo later, an embolic infarction causing right hemiparesis & aphasia |

Abbreviations: +, positive/present; & = and; ADC = apparent diffusion coefficient; CMV = Cytomegalovirus; CSF = cerebrospinal fluid; CT = computerized tomography; DWI = Diffusion-weighted imaging; F = Female; FLAIR = Fluid-attenuated inversion recovery; F-U = follow-up; GCS = Glasgow Outcome Scale; HIV = human immunodeficiency virus; IHC = immunohistochemistry; incl. = include; IV = intravenous; mo = month; M = Male; MRI = magnetic resonance imaging; NGS = next-generation sequencing; OR = operation; PCR = polymerase chain reaction; PMH = past medical history; PO = per os (by mouth); sp. = species; T1 = T1-weighted image; T2 = T2-weighted image; wk = week; yr = year.

*, cases reported in the last 50 years since CT was first used.

**, in cases with only CSF detection of pathogens, neuroimaging confirmed the brain intra-axial/parenchymal lesion(s).

**References:**

1. Kisiel M, Bass VM, Fong C, Graham AK, Yahya S, Eichorn FC, Lannon M, Kameda-Smith M, Reddy KKV, Lu JQ. Clinicopathologic characteristics of Nocardia brain abscesses: Necrotic and non-necrotic foci of various stages. *J Neurol Sci.* 2024;456:122850. doi: 10.1016/j.jns.2023.122850.
2. Lamtri Laarif M, Schils R, Lifrange F, Valkenborgh C, Pitti P, Brouwers P, Bianchi E, Meex C, Hayette MP. Actinomyces israelii and Fusobacterium nucleatum brain abscess in an immunocompetent patient: case report. *Access Microbiol.* 2023;5(6):acmi000499.v4. doi: 10.1099/acmi.0.000499.v4.
3. Chen YY, Xue XH. Coinfection of Streptococcus suis and Nocardia asiatica in the human central nervous system: A case report. *World J Clin Cases.* 2022;10(18):6283-6288. doi: 10.12998/wjcc.v10.i18.6283.
4. Wisutep P, Kamolvit W, Chongtrakool P, Jitmuang A. Brain abscess mimicking acute stroke syndrome caused by dual Filifactor alocis and Porphyromonas gingivalis infections: A case report. *Anaerobe*. 2022;75:102535. doi: 10.1016/j.anaerobe.2022.102535.
5. Altdorfer A, De Cassem J, Gavage P, Mathonet PY, Guzmán-Suárez S, Moerman F. A rare case of voluminous brain abscess due to Actinomyces meyeri and Aggregatibacter aphrophilus: is there any evidence for a prolonged antibiotic oral relay?. *J Infect Chemother*. 2021;27(8):1234-1237. doi: 10.1016/j.jiac.2021.02.005.
6. Graham AK, Sharma S, Yamamura D, Kameda-Smith MM, Main C, Lu JQ. Brain Toxoplasmosis and Bacterial Infection after Liver Transplantation. *Can J Neurol Sci*. 2023;50(1):155-157. doi: 10.1017/cjn.2021.494.
7. Ryu B, Khatri D, Zlochower A, Maslak S, D'Amico RS. Erosion of the sella turcica and pituitary expansion secondary to polymicrobial brain abscesses: a case report*. Access Microbiol.* 2021;3(10):000270. doi: 10.1099/acmi.0.000270.
8. Abo-Zed A, Yassin M, Phan T. A rare case of polymicrobial brain abscess involving *Actinomyces*. *Radiol Case Rep*. 2021;16(5):1123-1126. doi: 10.1016/j.radcr.2021.02.042.
9. Takahashi M, Nakanishi Y, Hamada Y, Hoshimoto Y, Aoki J, Karakida K. A Case of Brain Abscess Caused by Actinomyces Cardiffensis and Parvimonas Micra. *Tokai J Exp Clin Med.* 2020;45(4):189-194.
10. Rotter J, Graffeo CS, Perry A, Gilder HE, Wilson JW, Link MJ. Polymicrobial Intracerebral Abscess Growing Mycobacterium avium Complex and Achromobacter xylosoxidans: Case Report and Literature Review. *World Neurosurg.* 2020;141:441-447.e1. doi: 10.1016/j.wneu.2020.05.283.
11. Chandra V, Agarwal N, Zenonos GA, Zhang X, Hamilton RL, Gardner PA. Concomitant parasagittal meningioma and adjacent intracranial abscess of occult etiology. *J Clin Neurosci*. 2020;72:474-480. doi: 10.1016/j.jocn.2019.11.033.
12. Lo YP, Desale S, Wu PY. Brain abscess in a patient with psoriatic arthritis treated with adalimumab: A case report. *Medicine (Baltimore).* 2020;99(10):e18954. doi: 10.1097/MD.0000000000018954.
13. Shafquat Y, Zeeshan M, Chandio S, Mumtaz Z. Polymicrobial Cerebral Abscess in a Child with Uncorrected Tetralogy of Fallot. *J Pak Med Assoc.* 2019;69(9):1383-1384.
14. Frenchu KK, Ahmed MB. Brain Abscess in an Adult Patient With Congenital Heart Disease. *JAMA Cardiol*. 2019;4(2):184-185. doi: 10.1001/jamacardio.2018.3003.
15. Sood V, Pattanashetti N, Gupta S, Rudramurthy SM, Ramachandran R, Gupta KL. Multiple cerebral abscesses in a renal transplant recipient: Two swords in one scabbard!. *Med Mycol Case Rep.* 2018;23:50-52. doi: 10.1016/j.mmcr.2018.12.002.
16. Gonzales Zamora JA. Dual infection of the central nervous system caused by Cryptococcus and Toxoplasma in a patient with AIDS: a case report and literature review. *Acta Clin Belg*. 2018;73(6):448-452. doi: 10.1080/17843286.2018.1457761.
17. Nkamga VD, Lotte R, Chirio D, Lonjon M, Roger PM, Drancourt M, Ruimy R. Methanobrevibacter oralis detected along with Aggregatibacter actinomycetemcomitans in a series of community-acquired brain abscesses. *Clin Microbiol Infect.* 2018;24(2):207-208. doi: 10.1016/j.cmi.2017.08.021.
18. Chatzaraki V, Bolliger SA, Thali MJ, Eggert S, Ruder TD. Unexpected brain finding in pre-autopsy postmortem CT. *Forensic Sci Med Pathol*. 2017;13(3):367-371. doi: 10.1007/s12024-017-9880-7.
19. Mashiko R, Taguchi S, Tobita T, Shibata Y. Intracranial infection caused by minor skin contusion associated with previous craniotomy. *BMJ Case Rep*. 2017;2017:bcr2016217833. doi: 10.1136/bcr-2016-217833.
20. Srikumar T, Pabbathi S, Fernandez J, Nanjappa S. Aspergillus Terreus Brain Abscess Complicated by Tension Pneumocephalus in a Patient with Angiosarcoma. *Am J Case Rep.* 2017;18:33-37. doi: 10.12659/ajcr.900425.
21. Wang J, Fraser JF. An Intracranial Petri Dish? Formation of Abscess in Prior Large Stroke After Decompressive Hemicraniectomy. *World Neurosurg.* 2015;84(5):1495.e5-9. doi: 10.1016/j.wneu.2015.05.013.
22. Greninger AL, Langelier C, Cunningham G, Keh C, Melgar M, Chiu CY, Miller S. Two Rapidly Growing Mycobacterial Species Isolated from a Brain Abscess: First Whole-Genome Sequences of Mycobacterium immunogenum and Mycobacterium llatzerense. *J Clin Microbiol*. 2015;53(7):2374-7. doi: 10.1128/JCM.00402-15.
23. Bogdan M, Zujić Atalić V, Hećimović I, Vuković D. Brain abscess due to Aggregatibacter aphrophilus and Bacteroides uniformis. *Acta Med Acad*. 2015;44(2):181-5. doi: 10.5644/ama2006-124.144.
24. Su TM, Lan CM, Lee TH, Hsu SW. Gas-containing brain abscess: etiology, clinical characteristics, and outcome. *Kaohsiung J Med* Sci. 2014;30(12):619-24. doi: 10.1016/j.kjms.2014.10.003.
25. Jehangir W, Sareen R, Sen S, Raoof N, Yousif A. Acute Confusional State: A Manifestation of Toxoplasma and CMV Co-infection in HIV Patient. *N Am J Med Sci.* 2014;6(10):545-8. doi: 10.4103/1947-2714.143290.
26. Pallesen LP, Schaefer J, Reuner U, Leonhardt H, Engellandt K, Schneider H, Reichmann H, Puetz V. Multiple brain abscesses in an immunocompetent patient after undergoing professional tooth cleaning. *J Am Dent Assoc*. 2014;145(6):564-8. doi: 10.14219/jada.2014.20.
27. Sacks D, Kim E, Russell P. The role of the endonasal endoscope in the operative management of brain abscess: a case report. *J Neurol Surg A Cent Eur Neurosurg.* 2013;74 Suppl 1:e54-7. doi: 10.1055/s-0032-1330955.
28. Haane C, Krummenerl T, Matuszewski L, Rijcken E, Brüwer M, Neumann PA. Fatal complications in fistulizing Crohn's disease: brain abscess and squamous cell carcinoma. *Int J Colorectal Dis*. 2013;28(8):1171-3. doi: 10.1007/s00384-012-1607-3.
29. Larbcharoensub N, Chongtrakool P, Wirojtananugoon C, Watcharananan SP, Sumethkul V, Boongird A, Jirasiritham S. Treatment of a brain abscess caused by Scedosporium apiospermum and Phaeoacremonium parasiticum in a renal transplant recipient. *Southeast Asian J Trop Med Public Health.* 2013;44(3):484-9.
30. Johnson TJ, King C. An 11-year-old girl with right-sided weakness secondary to cerebral abscesses: a case report. *Pediatr Emerg Care*. 2013;29(3):360-3. doi: 10.1097/PEC.0b013e3182854645.
31. Simsek H, Kutlay M, Colak A, Haholu A, Kaya H, Ozyurt M, Demircan MN. Concomitant tubercular and fungal cerebellar abscess in an immunocompromised girl. *Turk Neurosurg*. 2013;23(1):88-94. doi: 10.5137/1019-5149.JTN.3718-10.1.
32. Azenha MR, Homsi G, Garcia IR Jr. Multiple brain abscess from dental origin: case report and literature review. *Oral Maxillofac Surg*. 2012;16(4):393-7. doi: 10.1007/s10006-011-0308-3.
33. Antunes AA, de Santana Santos T, de Carvalho RW, Avelar RL, Pereira CU, Pereira JC. Brain abscess of odontogenic origin. *J Craniofac Surg*. 2011;22(6):2363-5. doi: 10.1097/SCS.0b013e318231e585.
34. Hsu CW, Lu CH, Chuang MJ, Huang CR, Chuang YC, Tsai NW, Chen SF, Chang CC, Chang WN. Cerebellar bacterial brain abscess: report of eight cases. *Acta Neurol Taiwan.* 2011;20(1):47-52.
35. Stein M, Schirotzek I, Preuss M, Scharbrodt W, Oertel M. Brainstem abscess caused by Haemophilus influenza and Peptostreptococcus species. *J Clin Neurosci.* 2011;18(3):425-8. doi: 10.1016/j.jocn.2010.03.054.
36. Akhaddar A, Elouennass M, Baallal H, Boucetta M. Focal intracranial infections due to Actinomyces species in immunocompetent patients: diagnostic and therapeutic challenges. *World Neurosurg*. 2010;74(2-3):346-50. doi: 10.1016/j.wneu.2010.05.029.
37. Diaz M, Cuellar J, Valencia-Rey PA, Fisher JF. Unsuspected polymicrobial brain abscess arising from an intra-abdominal source in a patient with hereditary hemorrhagic telangiectasia. *South Med J.* 2010;103(8):842-4. doi: 10.1097/SMJ.0b013e3181e6d16e.
38. Keller PM, Rampini SK, Bloemberg GV. Detection of a mixed infection in a culture-negative brain abscess by broad-spectrum bacterial 16S rRNA gene PCR. *J Clin Microbiol*. 2010;48(6):2250-2. doi: 10.1128/JCM.01922-09.
39. Keiner D, Gaab MR, Ostertag H, Sommer C, Oertel J. Brain abscess formation within an endodermal cyst of the frontal lobe: case report. *Minim Invasive Neurosurg*. 2009;52(5-6):242-5. doi: 10.1055/s-0029-1239502.
40. Sakamoto H, Karakida K, Otsuru M, Arai M, Shimoda M. A case of brain abscess extended from deep fascial space infection. *Oral Surg Oral Med Oral Pathol Oral Radiol Endod.* 2009;108(3):e21-5. doi: 10.1016/j.tripleo.2009.05.002.
41. Shachor-Meyouhas Y, Guilburd JN, Kassis I. Brain abscess complicating foreign body aspiration. *Isr Med Assoc J.* 2009;11(9):564-5.
42. Ozüm U, Karadağ O, Eğilmez R, Engin A, Oztoprak I, Ozçelik S. A case of brain abscess due to Entamoeba species, Eikenella corrodens and Prevotella species. *Br J Neurosurg*. 2008;22(4):596-8. doi: 10.1080/02688690801894646.
43. Pham LV, Quang AT, Ton Nu PA, Duc TT, Thi HN. Cladophialophora bantiana and Candida albicans mixed infection in cerebral abscess of an HIV-negative patient. *J Infect Dev Ctries*. 2008;2(3):245-8. doi: 10.3855/jidc.271.
44. Roberts J, Bartlett AH, Giannoni CM, Valdez TA. Airway foreign bodies and brain abscesses: report of two cases and review of the literature. *Int J Pediatr Otorhinolaryngol*. 2008;72(2):265-9. doi: 10.1016/j.ijporl.2007.10.006.
45. Ramesh VG, Sundar KS. Concomitant tuberculous and pyogenic cerbellar abscess in a patient with pulmonary tuberculosis. *Neurol India*. 2008;56(1):91-2. doi: 10.4103/0028-3886.39326.
46. Yilmaz G, Aydin K, Bektas D, Caylan R, Caylan R, Koksal I. Cerebellar abscess and meningitis, caused by Shewanella putrefaciens and Klebsiella pneumoniae, associated with chronic otitis media. *J Med Microbiol.* 2007;56(Pt 11):1558-1560. doi: 10.1099/jmm.0.47044-0.
47. Kombogiorgas D, Seth R, Athwal R, Modha J, Singh J. Suppurative intracranial complications of sinusitis in adolescence. Single institute experience and review of literature. *Br J Neurosurg.* 2007; 21:603-609.
48. Young JP, Young PH. Meningioma associated with abscess formation--a case report. *Surg Neurol*. 2005;63(6):584-5. doi: 10.1016/j.surneu.2004.07.044.
49. Strojnik T, Roskar Z. Brain abscess after milk tooth self-extraction. *Wien Klin Wochenschr.* 2004;116 Suppl 2:87-9.
50. Friedlander RM, Gonzalez RG, Afridi NA, Pfannl R. Case records of the Massachusetts General Hospital. Weekly clinicopathological exercises. Case 16-2003. A 58-year-old woman with left-sided weakness and a right frontal brain mass. *N Engl J Med.* 2003;348(21):2125-32. doi: 10.1056/NEJMcpc030011.
51. Rao RP, Ghanayem NS, Kaufman BA, Kehl KS, Gregg DC, Chusid MJ. Mycoplasma hominis and Ureaplasma species brain abscess in a neonate. *Pediatr Infect Dis J.* 2002;21(11):1083-5. doi: 10.1097/00006454-200211000-00026.
52. Cihangiroglu M, Hartker FW, Mojtahadi S, Ramsey RG. Intracranial vasculitis and multiple abscesses in a pregnant woman. *J Neuroimaging.* 2001;11(3):340-2. doi: 10.1111/j.1552-6569.2001.tb00061.x.
53. Corson MA, Postlethwaite KP, Seymour RA. Are dental infections a cause of brain abscess? Case report and review of the literature. *Oral Dis.* 2001;7(1):61-5.
54. Yamamoto M, Fukushima T, Hirakawa K, Kimura H, Tomonaga M. Treatment of bacterial brain abscess by repeated aspiration--follow up by serial computed tomography. *Neurol Med Chir (Tokyo).* 2000;40(2):98-104; discussion 104-5. doi: 10.2176/nmc.40.98.
55. Lozniewski A, Maurer P, Schuhmacher H, Carlier JP, Mory F. First isolation of Desulfovibrio species as part of a polymicrobial infection from a brain abscess. *Eur J Clin Microbiol Infect Dis.* 1999;18(8):602-3. doi: 10.1007/s100960050357.
56. Koot RW, Reedijk B, Tan WF, De Sonnaville-De Roy Van Zuide. Neonatal brain abscess: complication of fetal monitoring. *Obstet Gynecol.* 1999;93(5 Pt 2):857. doi: 10.1016/s0029-7844(98)00372-x.
57. Sahjpaul RL, Lee DH. Infratentorial subdural empyema, pituitary abscess, and septic cavernous sinus thrombophlebitis secondary to paranasal sinusitis: case report*. Neurosurgery.* 1999;44(4):864-6; discussion 866-8. doi: 10.1097/00006123-199904000-00101.
58. Ruebenacker CA, Heary RF, Baredes S. Type A immunoglobulin deficiency presenting as a mixed polymicrobial brain abscess: case report. *Neurosurgery.* 1999;44(2):411-4. doi: 10.1097/00006123-199902000-00109.
59. Barlas O, Sencer A, Erkan K, Eraksoy H, Sencer S, Bayindir C. Stereotactic surgery in the management of brain abscess. *Surg Neurol* 1999; 52:404-410.
60. Akimura T, Ideguchi M, Kawakami N, Ito H. Brain abscess with fatal intraventricular rupture caused by asymptomatic paranasal sinusitis. *Eur Arch Otorhinolaryngol*. 1998;255(7):382-3. doi: 10.1007/s004050050083.
61. Maniglia RJ, Roth T, Blumberg EA. Polymicrobial brain abscess in a patient infected with human immunodeficiency virus. *Clin Infect Dis*. 1997;24(3):449-51. doi: 10.1093/clinids/24.3.449.
62. Tekkök IH, Baeesa SS, Higgins MJ, Ventureyra EC. Abscedation of posterior fossa dermoid cysts. *Childs Nerv Syst*. 1996;12(6):318-22. doi: 10.1007/BF00301020.
63. Simpson AJ, Das SS, Mitchelmore IJ. Polymicrobial brain abscess involving Haemophilus paraphrophilus and Actinomyces odontolyticus. *Postgrad Med J.* 1996;72(847):297-8. doi: 10.1136/pgmj.72.847.297.
64. Rhatigan MC, Taylor RH. A potentially life-threatening upper eyelid laceration. *Injury.* 1996;27(3):229-30. doi: 10.1016/0020-1383(95)00184-0.
65. Dethy S, Manto M, Kentos A, Konopnicki D, Pirotte B, Goldman S, Hildebrand J. PET findings in a brain abscess associated with a silent atrial septal defect. *Clin Neurol Neurosurg.* 1995;97(4):349-53. doi: 10.1016/0303-8467(95)00067-t.
66. Hall WA. Hereditary hemorrhagic telangiectasia (Rendu-Osler-Weber disease) presenting with polymicrobial brain abscess. Case report. *J Neurosurg*. 1994;81(2):294-6. doi: 10.3171/jns.1994.81.2.0294.
67. Navas E, Martínez-San Millán J, García-Villanueva M, de Blas A. Brain abscess with intracranial gas formation: case report. *Clin Infect Dis.* 1994;19(1):219-20. doi: 10.1093/clinids/19.1.219.
68. Case records of the Massachusetts General Hospital. Weekly clinicopathological exercises. Case 43-1993. A 71-year-old woman with confusion, hemianopia, and an occipital mass. *N Engl J Med.* 1993;329(18):1335-41. doi: 10.1056/NEJM199310283291809.
69. Case records of the Massachusetts General Hospital. Weekly clinicopathological exercises. Case 16-1990. A 57-year-old woman with a thalamic lesion and pulmonary arteriovenous fistulas. *N Engl J Med*. 1990;322(16):1139-48. doi: 10.1056/NEJM199004193221608.
70. Gray F, Gherardi R, Baudrimont M, Gaulard P, Meyrignac C, Vedrenne C, Poirier J. Leucoencephalopathy with multinucleated giant cells containing human immune deficiency virus-like particles and multiple opportunistic cerebral infections in one patient with AIDS. *Acta Neuropathol.* 1987;73(1):99-104. doi: 10.1007/BF00695508.
71. Bishburg E, Sunderam G, Reichman LB, Kapila R. Central nervous system tuberculosis with the acquired immunodeficiency syndrome and its related complex. *Ann Intern Med*. 1986;105(2):210-3. doi: 10.7326/0003-4819-105-2-210.
72. Chee CP, Coltheart GJ. Cerebritis preceding cerebral abscess formation: a report of three cases. *Aust N Z J Surg.* 1986;56(8):657-60. doi: 10.1111/j.1445-2197.1986.tb04523.x.
73. Stallworth JR, Perina D, Boykin D, Young FH Jr, Porter RC. Central nervous system nocardiosis associated with a traumatic polymicrobial brain abscess. *Pediatr Infect Dis.* 1985;4(4):411-3. doi: 10.1097/00006454-198507000-00018.
74. Dijkmans BA, Thomeer RT, Vielvoye GJ, Lampe AS, Mattie H. Brain abscess due to Streptobacillus moniliformis and Actinobacterium meyerii. *Infection*. 1984;12(4):262-4. doi: 10.1007/BF01645956.
75. Young RF, Frazee J. Gas within intracranial abscess cavities: an indication for surgical excision. *Ann Neurol.* 1984;16(1):35-9. doi: 10.1002/ana.410160108.
76. Press OW, Ramsey PG. Central nervous system infections associated with hereditary hemorrhagic telangiectasia. *Am J Med.* 1984;77(1):86-92. doi: 10.1016/0002-9343(84)90441-8.
77. Levy RM, Pons VG, Rosenblum ML. Central nervous system mass lesions in the acquired immunodeficiency syndrome (AIDS). *J Neurosurg.* 1984;61(1):9-16. doi: 10.3171/jns.1984.61.1.0009.
78. Grace A, Drake-Lee A. Role of anaerobes in cerebral abscesses of sinus origin. *Br Med J (Clin Res Ed)*. 1984;288(6419):758-9. doi: 10.1136/bmj.288.6419.758-a.
79. Sable NS, Hengerer A, Powell KR. Acute frontal sinusitis with intracranial complications. *Pediatr Infect Dis.* 1984;3(1):58-61. doi: 10.1097/00006454-198401000-00014.
80. Härkönen M. Hereditary hemorrhagic telangiectasia (Osler-Weber-Rendu disease) complicated by pulmonary arteriovenous fistula and brain abscess. *Acta Med Scand.* 1981;209(1-2):137-9. doi: 10.1111/j.0954-6820.1981.tb11567.x.
81. Warner JF, Perkins RL, Cordero L. Metronidazole therapy of anaerobic bacteremia, meningitis, and brain abscess. *Arch Intern Med.* 1979;139(2):167-9.
82. Román G, Fisher M, Perl DP, Poser CM. Neurological manifestations of hereditary hemorrhagic telangiectasia (Rendu-Osler-Weber disease): report of 2 cases and review of the literature. *Ann Neurol.* 1978;4(2):130-44. doi: 10.1002/ana.410040207.
83. Adams HP Jr, Subbiah B, Bosch EP. Neurologic aspects of hereditary hemorrhagic telangiectasia. Report of two cases. *Arch Neurol.* 1977;34(2):101-4. doi: 10.1001/archneur.1977.00500140055011.
